# Deciphering protein evolution and fitness landscapes with latent space models

**DOI:** 10.1038/s41467-019-13633-0

**Published:** 2019-12-10

**Authors:** Xinqiang Ding, Zhengting Zou, Charles L. Brooks III

**Affiliations:** 1 Department of Computational Medicine & Bioinformatics, University of Michigan, Ann Arbor, MI 48109 USA; 2 Department of Ecology and Evolutionary Biology, University of Michigan, Ann Arbor, MI 48109 USA; 3 Department of Chemistry, University of Michigan, Ann Arbor, MI 48109 USA; 4 Biophysics Program, University of Michigan, Ann Arbor, MI 48109 USA

**Keywords:** Biophysics, Machine learning, Phylogeny, Protein design

## Abstract

Protein sequences contain rich information about protein evolution, fitness landscapes, and stability. Here we investigate how latent space models trained using variational auto-encoders can infer these properties from sequences. Using both simulated and real sequences, we show that the low dimensional latent space representation of sequences, calculated using the encoder model, captures both evolutionary and ancestral relationships between sequences. Together with experimental fitness data and Gaussian process regression, the latent space representation also enables learning the protein fitness landscape in a continuous low dimensional space. Moreover, the model is also useful in predicting protein mutational stability landscapes and quantifying the importance of stability in shaping protein evolution. Overall, we illustrate that the latent space models learned using variational auto-encoders provide a mechanism for exploration of the rich data contained in protein sequences regarding evolution, fitness and stability and hence are well-suited to help guide protein engineering efforts.

## Abstract (web summary)

Multiple sequence alignments of proteins carry information about evolution, the protein’s fitness landscape and its stability in the face of mutations. Here, the authors demonstrate the utility of latent space models learned using variational autoencoders to infer these properties from sequences.

## Introduction

Advances in nucleic acid sequencing technology have yielded a large amount of protein sequence data as deposited in protein sequence databases such as UniProt^1^ and Pfam^2^. For many protein families, thousands of sequences from different species are available and these sequences can be aligned to construct multiple sequence alignments (MSAs)^2^. These naturally occurring diverse protein sequences in an MSA, belonging to a protein family but functioning in a diverse set of environments, are the result of mutation and selection occurring during the process of protein evolution. The selection in evolution favors sequences that have high fitness and filters out sequences that do not fold correctly or have low fitness. Therefore, it is expected that the distribution of sequences observed in extant species in an MSA carries information about a protein family’s properties, such as evolution^3^, fitness^4–6^, structure^3,7–13^, and stability^3,14–17^. Computational and theoretical methods that are able to infer these protein properties using the sequence data have proven to be useful tools for studying proteins^3–6,14,17^.

The current widely used method for inferring protein evolution with sequences is phylogeny reconstruction^18^. In phylogeny reconstruction, sequences are assumed to be generated by an amino acid substitution model and an unobserved phylogenetic tree, which represents the phylogenetic relationship between sequences. Given sequences, the major task in phylogeny reconstruction is to infer the phylogenetic tree using either maximum likelihood methods or Bayesian approaches^18,19^. Multiple algorithms for this purpose have been developed and are widely used in a number of applications^20–24^. Because of the discrete nature of trees and the vast number of possible tree structures for even just a few hundred sequences, searching for the true maximum likelihood tree is very challenging and computationally intensive. Most phylogeny reconstruction methods use heuristic approaches and do not scale to tens of thousands of sequences^24^. To infer phylogenetic relationships between tens of thousands of sequences, faster phylogeny reconstruction methods such as the FastTree^24^ have been developed. A common assumption made in phylogeny reconstruction methods is that, when sequences evolve based on the phylogenetic tree, each amino acid position in the protein evolves independently of other positions^18^. However, significant evidence suggests that high-order epistasis between two or more positions exists and plays an important role in shaping evolutionary trajectories^25^. These high-order epistasis effects are not taken into account by current phylogeny reconstruction methods.

A recent advance aimed at capturing epistasis between protein positions is the development of direct coupling analysis (DCA)^4,7,26–32^. In contrast to phylogeny reconstruction, DCA explicitly models second-order epistasis between pairs of positions by an energy-based probabilistic model. In the probabilistic model, epistasis is modeled as an interaction energy term between pairs of positions. Multiple studies have shown that the second-order epistasis inferred using DCA is highly correlated with physical side chain–side chain contacts in protein structures, which makes DCA a useful tool to predict protein residue contact maps from sequences^4,7,11–13,26–32^. However, because DCA methods model the distribution of sequences directly instead of assuming that there is an underlying latent process generating the sequences as in phylogeny reconstruction, DCA methods cannot infer phylogenetic relationships between sequences. Moreover, because DCA methods aim to distinguish correlations caused by protein structure or function constraints from that caused by phylogeny, DCA methods implicitly reduce phylogenetic effects as suggested in ref. ^33^. In addition, the approach used by DCA to model second-order epistasis cannot be readily extended to model higher-order epistasis because the number of parameters in DCA models increases exponentially with the order of epistasis accounted for in the model. A DCA model with third-order epistasis would have too many parameters to fit given current sequence availability.

In this paper, we explore the application of latent space generative models^34,35^ on protein sequences to address limitations of both phylogeny reconstruction and DCA methods. Similarly to phylogeny reconstruction, the employed latent space model also assumes that protein sequences are generated from an underlying probabilistic generative process. However, the latent variables are continuous variables instead of tree structures. In contrast to DCA, the latent space model can theoretically model high-order epistasis without exponentially increasing the number of parameters, because the epistasis effect is modeled through latent variables. Learning the latent space model with a large amount of data is challenging and it has been an intensive research topic in both statistical inference and machine learning^36^. Thanks to recent advances in stochastic variational inference such as the variational auto-encoder (VAE) approach^34,35^, continuous latent space models can be readily learned for hundreds of thousands of sequences. All latent space models in this study were learned using the VAE approach.

With examples of both natural protein families and simulated sequences, we show that the continuous latent space model trained with VAEs can work beyond the limitations of previous methods. The latent space variable can capture evolutionary relationships, including ancestral relationships between sequences. In addition to modeling evolution, the latent space model also provides a continuous low-dimensional space in which protein fitness landscapes can be modeled. Moreover, we also find that the sequence probability assigned by the model is useful in predicting protein stability change upon mutations. The correlation between sequence probability change and protein stability change upon mutations provides an estimate of the importance of protein stability in protein evolution. Our findings suggest that, with the continuing increase in the amount of protein sequence data, latent space generative models trained with VAEs will be useful tools for both the study and engineering of proteins.

Learning latent space models of protein families using VAEs has also been explored by several other groups^37–39^, but the focus of applications presented in this study is different from that in previous studies. For instance, one of our findings that the latent space model trained with VAEs can capture phylogenetic relationships has not been investigated before. Modeling protein fitness landscapes in the latent space is also absent in previous studies^37–39^. A detailed comparison of our approach with previous studies is included in the Discussion section.

## Results

### Latent space models of protein MSAs

**Fig. 1:**
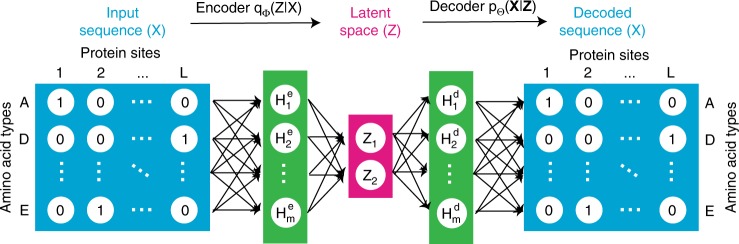
Encoder and decoder models used in variational auto-encoders. Both encoder and decoder models used in this paper are fully connected artificial neural networks with one hidden layer ${\bf{H}}$. The encoder model transforms each protein sequence ${\bf{X}}$ into a distribution ${q}_{{\boldsymbol{\phi }}}({\bf{Z}}| {\bf{X}})$ of ${\bf{Z}}$ in the latent space; the decoder model transforms each point in the latent space ${\bf{Z}}$ into a distribution ${p}_{{\boldsymbol{\theta }}}({\bf{X}}| {\bf{Z}})$ of ${\bf{X}}$ in the protein sequence space. Protein sequences from a multiple sequence alignment with $L$ amino acids are represented as a $21\times L$ matrix whose entries are either 0 or 1 based on a one-hot coding scheme. Gaps in sequences are modeled as an extra amino acid type. Therefore, there are 21 amino acid types.

The protein sequences in a protein family’s MSA are the result of mutation and selection occurring during the process of protein evolution. Therefore, it is expected that the distribution of sequences observed in extant species in an MSA carries information about the protein family’s properties, such as its evolution^3^. It is through modeling the sequence distribution of a protein family that latent space models infer evolution and other properties. In latent space models, a protein sequence ${\bf{S}}=({s}_{1},{s}_{2},...,{s}_{L})$ from an MSA with $L$ positions is represented as a binary $21\times L$ matrix ${\bf{X}}$ for which ${X}_{ij}=1$ if ${s}_{j}=i$ and otherwise ${X}_{ij}=0$ (Fig. 1). (${s}_{j}$ corresponds to the amino acid type at the $j$th position of the protein and amino acid types are labeled using numbers from 0 to 20, where 0 represents a gap in the MSA and numbers 1 to 20 represent the 20 natural amino acid types.)

In addition to the variables ${\bf{X}}$ representing sequences, latent space models also include latent variables ${\bf{Z}}$ and the generative process ${p}_{{\boldsymbol{\theta }}}({\bf{X}}| {\bf{Z}})$. Latent variables ${\bf{Z}}$ can be viewed as a code for ${\bf{X}}$. Latent space models define the joint distribution of ${\bf{X}}$ and ${\bf{Z}}$ as ${p}_{{\boldsymbol{\theta }}}({\bf{X}},{\bf{Z}})={p}_{{\boldsymbol{\theta }}}({\bf{Z}}){p}_{{\boldsymbol{\theta }}}({\bf{X}}| {\bf{Z}})$, where ${\boldsymbol{\theta }}$ represents parameters of the joint distribution. The joint distribution ${p}_{{\boldsymbol{\theta }}}({\bf{X}},{\bf{Z}})={p}_{{\boldsymbol{\theta }}}({\bf{Z}}){p}_{{\boldsymbol{\theta }}}({\bf{X}}| {\bf{Z}})$ implies a probabilistic generative process for $({\bf{X}},{\bf{Z}})$: the latent variables ${\bf{Z}}$ are sampled from a prior distribution ${p}_{{\boldsymbol{\theta }}}({\bf{Z}})$ first and then the sequence variables ${\bf{X}}$ are sampled from the conditional distribution ${p}_{{\boldsymbol{\theta }}}({\bf{X}}| {\bf{Z}})$ given ${\bf{Z}}$. The conditional distribution ${p}_{{\boldsymbol{\theta }}}({\bf{X}}| {\bf{Z}})$ can also be viewed as a decoder that converts codes ${\bf{Z}}$ into protein sequences ${\bf{X}}$. Although protein sequences ${\bf{X}}$ are discrete random variables, the latent space variables ${\bf{Z}}$ are modeled as continuous random variables.

Given the observed sequence data for variables ${\bf{X}}$, learning the parameters ${\boldsymbol{\theta }}$ that describe the generative process using maximum likelihood approaches is challenging and has been an intensive research topic in machine learning^34,36^. One reason for the difficulty is that the marginal probability of the observed sequences ${\bf{X}}$,

1 $${p}_{{\boldsymbol{\theta }}}({\bf{X}})=\int {p}_{{\boldsymbol{\theta }}}({\bf{X}},{\bf{Z}}){\rm{d}}{\bf{Z}},$$

is not analytically tractable and is expensive to compute when the conditional distribution ${p}_{{\boldsymbol{\theta }}}({\bf{X}}| {\bf{Z}})$ is complex. The other reason for the difficulty is that when the conditional distribution ${p}_{{\boldsymbol{\theta }}}({\bf{X}}| {\bf{Z}})$ is complex, such as parameterized by an artificial neural network, the posterior distribution ${p}_{{\boldsymbol{\theta }}}({\bf{Z}}| {\bf{X}})$ becomes analytically intractable. Moreover, it can also be difficult to efficiently draw independent samples from ${p}_{{\boldsymbol{\theta }}}({\bf{Z}}| {\bf{X}})$^34^, which makes the expectation-maximization algorithm^40,41^ unsuitable for maximizing the marginal probability ${p}_{{\boldsymbol{\theta }}}({\bf{X}})$. One effective way to learn the parameters ${\boldsymbol{\theta }}$ is to use an approximation method called variational inference^36,42,43^. In variational inference, to remedy the difficulty with the posterior distribution ${p}_{{\boldsymbol{\theta }}}({\bf{Z}}| {\bf{X}})$, a family of approximate distributions, ${q}_{{\boldsymbol{\phi }}}({\bf{Z}}| {\bf{X}})$, parameterized by ${\boldsymbol{\phi }}$, is introduced to approximate the posterior distribution ${p}_{{\boldsymbol{\theta }}}({\bf{Z}}| {\bf{X}})$. Instead of optimizing the marginal probability of observed sequences ${p}_{{\boldsymbol{\theta }}}({\bf{X}})$, variational inference optimizes an alternative objective function called the evidence lower bound objective function (ELBO)^34,36^, which is defined as

2 $${\rm{ELBO}}({\boldsymbol{\theta }},{\boldsymbol{\phi }})= \sum _{{\bf{Z}}}{q}_{{\boldsymbol{\phi }}}({\bf{Z}}| {\bf{X}}){\mathrm{log}}\,{p}_{{\boldsymbol{\theta }}}({\bf{X}}| {\bf{Z}}) - \sum _{{\bf{Z}}}{{q}}_{{\boldsymbol{\phi }}}({\bf{Z}}| {\bf{X}}){\mathrm{log}}\frac{{q}_{{\boldsymbol{\phi }}}({\bf{Z}}| {\bf{X}})} {{p}_{{\boldsymbol{\theta }}}({\bf{Z}})},$$

where the first term represents the model’s reconstruction power from the latent space representation and the second term is the Kullback–Leibler divergence between the approximation distribution ${q}_{{\boldsymbol{\phi }}}({\bf{Z}}| {\bf{X}})$ and the prior distribution ${p}_{{\boldsymbol{\theta }}}({\bf{Z}})$. It can be easily proved that the ELBO objective function is a lower bound of the log likelihood function, i.e., ${\rm{ELBO}}({\boldsymbol{\theta }},{\boldsymbol{\phi }})\ \le \ {\mathrm{log}} \, {p}_{{\boldsymbol{\theta }}}({\bf{X}})$^36,42^.

Two recent advances that enable variational inference approaches to learn latent space models for a large amount of data are stochastic variational inference^44^ and VAEs^34,35^. VAEs combine stochastic variational inference with a reparameterization strategy for the amortized inference model ${q}_{{\boldsymbol{\phi }}}({\bf{Z}}| {\bf{X}})$^34,35^. Latent space models learned with VAEs have been widely used in several machine learning problems, such as image and natural language processing, and produce state-of-the-art results^34,45,46^. In this study, we utilize the VAE approach to learn latent space models of MSAs of protein families. Specifically, the prior distribution of ${\bf{Z}}$, ${p}_{{\boldsymbol{\theta }}}({\bf{Z}})$, is chosen to be a multivariable normal distribution with a mean of zero and an identity covariance. The encoder conditional distribution ${q}_{{\boldsymbol{\phi }}}({\bf{Z}}| {\bf{X}})$ and the decoder conditional distribution ${p}_{{\boldsymbol{\theta }}}({\bf{X}}| {\bf{Z}})$ are parameterized using artificial neural networks with one hidden layer (Fig. 1), similarly to the model used in the original VAE paper^34^.

### Latent space representations capture phylogeny

The encoder ${q}_{{\boldsymbol{\phi }}}({\bf{Z}}| {\bf{X}})$, trained on the MSA of a protein family, can be used to embed sequences in a low-dimensional continuous latent space, ${\bf{Z}}$, i.e., each sequence from the MSA is projected into a point in the latent space. Embedding sequences in a low-dimensional continuous space can be useful for several reasons. The low (2 or 3) dimensionality makes it straightforward to visualize sequence distributions and sequence relationships. The continuity of the space enables us to apply operations such as interpolation and extrapolation, which are best suited to continuous variables, to the family of sequences, and this, in turn, can allow us to explore new sequences through decoding the relationships implied by the MSA.

**Fig. 2:**
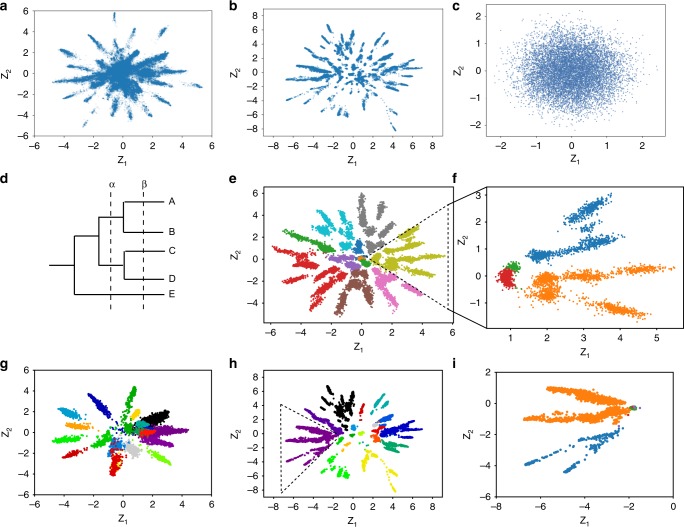
Latent space representation of sequences captures phylogenetic relationships between sequences. **a**, **b** Latent space representation of sequences from the multiple sequence alignment of the fibronectin type III domain and the cytochrome P450 family, respectively. **c** Latent space representation of 10,000 random sequences with 100 amino acids sampled from the equilibrium distributions of the LG evolutionary model. **d** A schematic representation of the phylogenetic tree used to simulate the evolution of a random protein sequence with 100 amino acids. The actual tree has 10,000 leaf nodes. The dashed lines, $\alpha$ and $\beta$, represent two reference evolutionary time points on which sequences of leaf nodes are grouped. Sequences of leaf nodes are in the same group if they are in the same branch at the reference time point, either $\alpha$ or $\beta$, which have an evolutionary distance of 0.5 and 0.9 from the root node, respectively. The evolutionary distance from the root node represents the expected number of substitutions per site compared to the root node sequence. **e** Latent space representation of simulated sequences of all leaf nodes. Sequences are separated into groups at the reference time point $\alpha$. Sequences are colored based on groups. Quantification of the clustering can be found in Supplementary Fig. 4. **f** Sequences from the yellow colored group (enclosed by the dashed triangle) in **e** are regrouped and recolored based on the reference time point $\beta$. **g** Latent space representation of grouped sequences of the fibronectin type III domain family. A phylogenetic tree is inferred based on its MSA using FastTree2. Based on the inferred phylogenetic tree, sequences are grouped similarly as in **d**, **e** with an evolutionary distance of 2.4. The top 20 largest groups of sequences are plotted and sequences are colored based on their group. **h** A similar plot as **g** for the cytochrome P450 family. **i** Sequences from the purple colored group (enclosed by the dashed triangle) in **h** are regrouped and recolored based on a reference time point with an evolutionary distance of 2.6.

To see how sequences from such an MSA are distributed in the latent space, we trained latent space models using VAEs on MSAs from three protein families: fibronectin type III domain (Pfam accession id: PF00041), cytochrome P450 (PF00067), and staphylococcal nuclease (PF00565). The number of unique sequences used for training the latent space models was 46,498, 31,062, and 7448, respectively. For visualization purposes, a two-dimensional latent space is used. Utilizing the learned encoder ${q}_{{\boldsymbol{\phi }}}({\bf{Z}}| {\bf{X}})$, sequences from MSAs are projected into the two-dimensional latent space ${\bf{Z}}$ for all three protein families (Fig. 2a, b, and Supplementary Fig. 1A). Results from Fig. 2a, b and Supplementary Fig. 1A show that, in the latent space, sequences are not distributed randomly. Their distributions have a star structure with multiple spikes, each of which points from the center toward the outside along a specific direction. As a negative control, the same latent space model is trained on an MSA consisting of 10,000 random sequences sampled from the equilibrium distribution of the LG evolutionary model^47^. In contrast to sequences from the above three natural protein families, these random sequences are randomly distributed in the latent space and the star structure is not observed (Fig. 2c). The difference between random sequences and sequences from a protein family’s MSA is that the latter are evolutionarily related. Therefore, the star structure observed in the latent space representation arises from evolutionary relationships between protein sequences in an MSA.

In evolution biology, the evolutionary relationship between sequences is often represented using a phylogenetic tree. To explore whether and how the latent space representation is related to phylogenetic relationships between sequences, we need to know the phylogenetic tree structures for sequences from the natural protein families (Fig. 2a, b and Supplementary Fig. 1A). Unfortunately, the phylogenetic tree structures cannot be known exactly for natural protein families. Therefore, to further explore whether and how the latent space representation captures phylogenetic relationships between sequences, we compared latent space representations with phylogenetic trees under three different scenarios: (1) simulated MSAs based on a random phylogenetic tree, (2) simulated MSAs based on realistic phylogenetic trees of natural protein families, and (3) natural protein MSAs with inferred phylogenetic trees. These three scenarios will be henceforth referred to as the first, second, and third scenarios, respectively. In the first and second scenarios with simulated protein sequences, the amino acid preferences of each protein site is independent from other sites, whereas in the third scenario with natural protein sequences, the amino acid preferences of each site include both site-specific effects and co-evolution effects between sites.

In the first scenario, a simulated MSA was generated by neutrally evolving a random protein sequence with 100 amino acids on a simulated phylogenetic tree^48^ with 10,000 leaf nodes and combining sequences from all the leaf nodes (Fig. 2d). Thus the phylogenetic relationships between sequences in this simulated MSA are known based on the phylogenetic tree defined in the simulation. As with the three natural protein families, the latent space representation of the simulated sequences has a similar star structure with multiple separate spikes (Fig. 2e). Although sequences in both Fig. 2c, e are from simulations, the star structure only appears in Fig. 2e, where sequences are simulated based a phylogenetic tree. This again supports the idea that the star structure is derived from evolutionary relationships encoded in the tree structure. To compare the latent space star structure with the phylogenetic tree, sequences are grouped together if they are in the same branch at a reference evolutionary time point ($\alpha$ and $\beta$ in Fig. 2d) based on the phylogenetic tree. Sequences in the same group have the same color in their latent space representation (Fig. 2e). Sequences with the same color are observed to have their latent space representations in the same spike or multiple adjacent spikes (Fig. 2e). The multiple adjacent spikes occupied by the same group of sequences represent more fine-grained phylogenetic relationships between sequences. These more fine-grained phylogenetic relationships can be recovered by changing the reference time point to $\beta$ to group the sequences (Fig. 2f).

In the second scenario, simulated MSAs were generated by evolving sequences on realistic phylogenetic trees of natural protein families. Seven realistic phylogenetic trees from the benchmark set of the FastTree study^49^ were used (http://www.microbesonline.org/fasttree/downloads/aa5K_new.tar.gz). Each of the seven realistic phylogenetic trees has 5000 leaf nodes. They were constructed using PhyML^50^ based on alignments of seven protein families from the Clusters of Orthologous Groups (COG) database. MSAs with 5000 sequences and 100 amino acids were simulated based on these realistic phylogenetic trees. As in the first scenario, the latent space representations of simulated sequences based on realistic phylogenetic trees also have star structures with multiple separate spikes (Supplementary Figs. 2 and 3). Because the phylogenetic trees underlying the simulations are known, we can also group sequences based on their evolutionary relationship by choosing an evolutionary distance threshold. As in the first scenario, we also observe that sequences belonging to the same group are clustered together in one spike or multiple adjacent spikes (Supplementary Figs. 2 and 3).

In the third scenario, approximate phylogenetic trees for the three protein families (fibronectin type III domain, cytochrome P450, and staphylococcal nuclease) were inferred using FastTree 2^49^. Then the sequences were grouped based on inferred phylogenetic trees. As shown in Fig. 2g–i and Supplementary Fig. 1B, real protein sequences from the same group are also embedded closely in the latent space, either in one spike or multiple adjacent spikes.

In summary, under all three different scenarios, the spatial organization of the latent space representation captures features of the phylogenetic relationship between sequences from an MSA of a protein family. To quantify the extent to which phylogenetic relationships between sequences can be captured by their latent space representations and how this changes with respect to the dimension of the latent space, the following analysis was conducted in the first scenario. Using the latent space representation, sequences are hierarchically clustered^51^. The Euclidean distance in the latent space was used as distance between sequences and the Ward’s minimum variance method^51^ was used as distance between clusters. Hierarchical clustering builds a tree structure of the sequences with all the sequences as its leaf nodes. Given a tree structure with sequences as its leaf nodes, sequences can be clustered at different resolutions by cutting the tree at different locations. For example, cutting the tree in Fig. 2d at the $\alpha$ and $\beta$ positions will generate clustering of sequences at two different resolutions, i.e., ((A,B), (C,D), (E)) with three clusters and ((A), (B), (C), (D), (E)) with five clusters. Because the underlying phylogenetic tree for the simulated MSAs is known in the first scenario, the true clustering of sequences at different resolutions is known based on the phylogenetic tree. Therefore, we can use the agreement between the true clustering and the hierarchical clustering result, which is based on latent space representations, to quantify how well latent space representations capture phylogenetic relationships. The agreement is calculated for clustering at different resolutions and is quantified using the widely used clustering comparison metric, the adjusted mutual information (AMI)^52^. To compare with traditional phylogenetic reconstruction methods, we also calculated the AMI between the true clustering and the clustering results based on the inferred phylogenetic tree using the FastTree 2^49^. Results of ten independent repeating experiments are shown in Supplementary Fig. 4. The performance of the clustering based on latent space representation increases when the dimension of latent space increases from 2 and becomes flat before the dimension increases to 20. Compared with FastTree 2, the clustering based on latent space representations usually has better performance at low clustering resolution, i.e., when the number of clusters is relatively small (less than a few hundreds of clusters for 10,000 sequences). At high clustering resolution, the performance of FastTree 2 is better than the clustering based on latent space representations. Therefore, compared with FastTree 2, the latent space representation is better at capturing low-resolution phylogenetic relationships and is worse at capturing high-resolution phylogenetic relationships. However, we note that FastTree 2 uses more prior information than do latent space models, such as the amino acid evolutional model and an out-of-group sequence, which is used for rooting the inferred phylogenetic tree. Neither of these is needed in learning latent space models. In addition, using more intricate metrics than Euclidean distance and other clustering methods might further improve the clustering performance of latent space models, which is the topic of future studies.

Because the dimension of the latent space is much smaller than that of the original sequence space, the VAE encoder can be viewed as a dimension reduction method for protein sequences. To test whether other dimension reduction methods can capture phylogenetic relationships between sequences as does the latent space model, we applied two widely used dimensional reduction methods, principal component analysis (PCA)^53^ and t-SNE^54^, to the same set of simulated sequences from Fig. 2e and embedded these sequences in the corresponding two-dimensional space (Supplementary Fig. 5). Sequences in Supplementary Fig. 5 are colored similarly as in Fig. 2e. In PCA, the first two components can only explain 3% of the variance observed in the original sequences and sequences belonging to different phylogenetic tree branches are overlapped with each other (Supplementary Fig. 5A). For t-SNE, although sequences belonging to different phylogenetic tree branches are not overlapped in the embedding space, they are not well separated, i.e., sequences from different branches are clustered together (Supplementary Fig. 5B). In addition, sequences from the same branch are separated into small clusters that are far apart in the embedding space (Supplementary Fig. 5B). Therefore, the phylogenetic relationships captured by the latent space model cannot be obtained or are more obscured using either PCA or t-SNE.

### Ancestral relationships present in latent space models

**Fig. 3:**
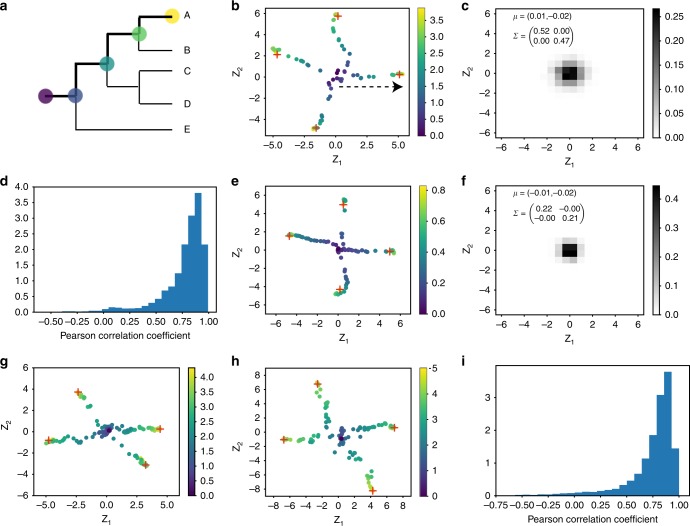
Latent space representation of sequences captures ancestral relationship between sequences. **a**–**d** Results for simulated MSAs based on random phylogenetic trees: **a** A schematic representation of the phylogenetic tree used to simulate the evolution of a random protein sequence with 100 amino acids. It is the same tree as in Fig. 2d. Here the evolutionary trace from the root node to the leaf node A is highlighted as bold lines. Nodes along the highlighted evolutionary trace are colored based on the evolutionary distance from the root node using the color bar shown in **b**. **b** Latent space representation of four representative leaf node sequences, labeled as plus signs, and their ancestral sequences, labeled as dots. Sequences are colored based on their evolutionary distances from the root node. The sequence of the root node sits around the origin in the latent space. As the sequence evolves from the root node to a leaf node, its latent space representation moves from the origin toward the surroundings along a direction. The moving direction, labeled as a dashed arrow line for the right most leaf node, is calculated as the first component direction using principal component analysis. **c** The distribution of the root node sequence position in the latent space estimated using 2000 repeats. **d** As shown in **b**, evolutionary distances of sequences are correlated with their positions along the first component direction in the latent space. The corresponding Pearson correlation coefficient can be calculated for each leaf node (see Supplementary Fig. 6A for the right most leaf node in **b**). Here we show the distribution of Pearson correlation coefficients of all leaf node sequences. **e**, **f** Results on simulated MSAs based on the realistic phylogenetic tree of COG642: **e** A similar plot as **b** for the COG642 family. **f** A similar plot as **c** for the COG642 family. **g**, **h** Similar plots as **b** for the fibronectin type III domain (**g**) and the cytochrome P450 family (**h**), respectively. **i** A similar plot as **d** for the fibronectin type III domain family.

Similarly to the manner that branches in phylogenetic trees share a common root node, spikes in latent space star structures share a common point near the origin of the latent space. This similarity is first supported by the observation that latent space representations of root node sequences tend to be near the origin of the latent space under all three different scenarios (Fig. 3b, e, g, h). To quantify the robustness of this observation and to examine how close root node sequence positions are to the origin, we conducted the following independent iterated calculation to estimate the uncertainty of the root node sequence position under the three scenarios explored above.

In the first scenario, the calculation was repeated 2000 times. In each repeat, a random phylogenetic tree with 10,000 leaf nodes was sampled and used to simulate an MSA with 100 amino acids. Then a latent space model was trained on the simulated MSA with the VAE. Sequences from both the root node and all leaf nodes were projected into the latent space with the learned encoder of the VAE. The overall range of leaf node sequence positions is from −6.5 to 6.5 along both ${z}_{1}$ and ${z}_{2}$. Figure 3c shows the empirical distribution of the root node sequence’s position in the latent space estimated using the 2000 repeats stated above. The mean of the empirical distribution is $(0.01,-0.02)$. The variances along ${z}_{1}$ and ${z}_{2}$ are $0.52$ and $0.47$, respectively. The distributions of distances from the origin for the root sequences and the sequences in the alignments (sequence on the leaf nodes) are plotted in Supplementary Fig. 7. As shown in Supplementary Fig. 8, similar results regarding the position of the root node sequence are also observed for simulations with heterotachy, where the substitution rate of each site changes over time. Therefore, on average in the first scenario, the root node sequence’s position in latent space is around the origin with a standard deviation of about $0.7$. In the second scenario, a similar calculation was conducted for each COG protein family as in the first scenario, except that the same realistic phylogenetic tree of the COG protein family was used across repeats. Results are shown in Fig. 3f and Supplementary Figs. 9 and 10. For all seven COG protein families, the overall range of leaf node sequence positions is from −6.5 to 6.5 and the means of empirical distributions of root node sequences’ positions are also close to the origin (Fig. 3f and Supplementary Figs. 9 and 10). The standard deviation is about $0.7$ for three of COG protein families and about $0.45$ for the other four COG protein families. In the third scenario, the inferred phylogenetic tree and sequences were fixed in each repeat and the latent space model was independently trained. For all three natural protein families, the mean of the root node sequence’s position is also close to origin (Supplementary Fig. 11). The standard deviations are 0.17, 1.01, and 1.40 for fibronectin type III, cytochrome P450, and staphylococcal nuclease protein family, respectively (Supplementary Fig. 11). The standard deviation is inversely correlated with the number of unique sequences used to train the latent space model.

Furthermore, to visualize how a sequence’s representation changes in latent space as the sequence evolves from the root node to a leaf node, we projected both leaf node sequences and their corresponding ancestral sequences into the latent space. Figure 3b shows the latent space representation of four example leaf node sequences and their ancestral sequences colored based on their evolutionary distance. We observed that, as sequences evolve from the root node to a leaf node, their positions in the latent space move from near the origin toward the outside along a direction. For a leaf node sequence and its corresponding ancestral sequences, the primary direction of motion is calculated as the first component direction using PCA (Fig. 3b). It is observed that a sequence’s distance from the origin along the moving direction in the latent space is highly correlated with the sequence’s evolutionary distance from the root node sequence (The Pearson correlation coefficient calculated using the right most leaf node sequence in Fig. 3b is 0.98 as shown in Supplementary Fig. 6A.). This correlation suggests that as sequences evolve from the root node toward leaf nodes in the phylogenetic tree, their latent space representations move from the origin of the latent space toward the outside along specific directions (Fig. 3b). This pattern holds for most of the leaf node sequences and their corresponding ancestral sequences (Fig. 3d). Similar results were also observed in the second and third scenarios (Fig. 3e, g–i and Supplementary Figs. 6, 9, and 10).

Because the prior distribution ${p}_{{\boldsymbol{\theta }}}({\bf{Z}})$ is symmetric with respect to rotation of the latent space and the regularization with Frobenius norm is symmetric with respect to the rotation of weights, the ELBO objective function (Eq. (2)) is symmetric to the rotation of mapping between the latent space and the hidden layer in the encoder model when the mapping between the latent space and the hidden layer in the decoder model is simultaneously inversely rotated. Therefore, rotating the latent space representation, calculated with the encoder model learned by optimizing the ELBO (Eq. (2)), by an arbitrary angle would yield an equally good encoder model in terms of the ELBO value. Consistent with this rotational symmetry, the star structure of sequences in latent space capturing the phylogenetic relationship is also invariant with respect to the rotation of latent space. The rotational symmetry of the latent space representation is also consistent to and closely related to the observation that, as a sequence evolves, its latent space representation moves from the origin toward the outside along a spike.

### Navigating protein fitness landscapes in latent space

The protein fitness here refers to protein properties contributing to the normal functioning of a protein, not the typical organismal fitness concept used in evolution biology. A protein’s fitness landscape is a map from the protein’s sequence to the protein’s fitness, such as the protein’s stability and activity, among a host of other properties. Knowing a protein’s fitness landscape can greatly assist in studying and engineering proteins with altered properties. A protein’s fitness landscape can also be viewed as a fitness function in a high-dimensional discrete space of sequences. Because of the high dimensionality and discreteness of this sequence space, and the effects of epistasis between different protein positions, it has been difficult for protein researchers to characterize protein fitness landscapes^25^. As only a relatively small number of sequences can be synthesized and have experimentally measured fitness values, a common problem facing researchers is, given the fitness values for a small collection of sequences from a protein family, how does one predict the fitness value of a new sequence from the same protein family or design a new sequence, which will have a desired fitness value.

Here we examine the use of a semi-supervised learning framework utilizing the latent space representation to learn protein fitness landscapes using both protein sequence data and experimental fitness data. Although fitness values are usually known for only a small subset of sequences from a protein family, we often have access to a large number of homologous sequences from the same protein family. These sequences represent functional proteins from species living in different environments. The distribution of these sequences is shaped by evolutionary selection. Therefore, we expect that the distribution of these sequences contains information about the relationship between sequence and fitness. To utilize this information, with a large number of sequences from a protein family, we can model the distribution of sequences by learning a latent space model for the protein family. The resulting latent space model trained using VAEs provides us with a sequence encoder and a sequence decoder. With the sequence encoder, sequences are first embedded into a low-dimensional continuous latent space. Then the fitness landscape is modeled in the latent space with experimental fitness data. With an estimated fitness landscape in the latent space, we can predict the fitness value of a new sequence using its latent space representation. In addition, we can also design new sequences with desired fitness values by choosing points in the latent space based on the fitness landscape and converting these points into sequences using the decoder. To test this framework, we applied it to the cytochrome P450 protein family (PF00067)^55–57^.

The cytochrome P450 protein family was chosen to test our framework because both experimental fitness data and a large number of sequences are available for this protein family. The Arnold group made a library of 6561 chimeric cytochrome P450 sequences by recombining three cytochrome P450s (CYP102A1, CYP102A2, CYP102A3) at seven crossover locations^55^ (Supplementary Fig. 12) and measured ${T}_{50}$ values (the temperature at which 50% of the protein is inactivated irreversibly after 10 min) for 278 sequences (Supplementary Table 1 and Supplementary Data 1)^55–57^. In addition to these experimental ${T}_{50}$ fitness data, the cytochrome P450 family has >31K unique homologous sequences in its MSA from the Pfam database^2^.

**Fig. 4:**
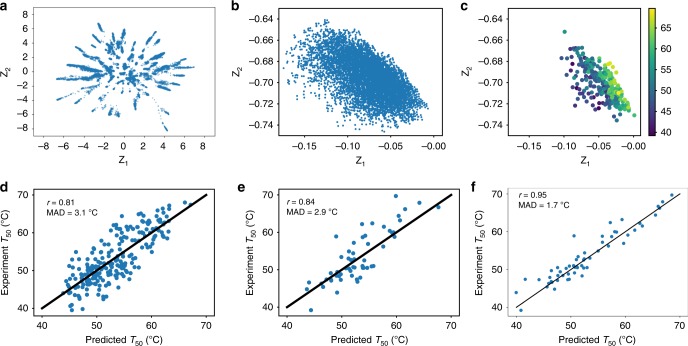
Navigating the protein fitness landscape in the VAE latent space. **a** A two-dimensional latent space representation of sequences from the cytochrome P450 family (PF00067). **b** The two-dimensional latent space representation of 6561 chimeric cytochrome P450 sequences made by combining the three cytochrome P450s (CYP102A1, CYP102A2, CYP102A3) at seven crossover locations. **c** The two-dimensional latent space representation of 278 chimeric cytochrome P450 sequences whose ${T}_{50}$ values were measured experimentally by the Arnold group^55–57^. Each point represents a chimeric cytochrome P450 sequence. Points are colored by their experimental ${T}_{50}$ values. **d** The Gaussian process’s performance at predicting ${T}_{50}$ on the training set of 222 chimeric cytochrome P450 sequences using the two-dimensional latent space representation (${Z}_{1}$, ${Z}_{2}$) as features and using the radial basis function kernel with Euclidean distance in latent space *Z*. **e** The performance of the Gaussian process model from **d** at predicting *T*_50_ on the test set of 56 chimeric cytochrome P450 sequences. **f** The Gaussian process’s performance at predicting ${T}_{50}$ on the test set of 56 chimeric cytochrome P450 sequences using the 20-dimensional latent space representation (${Z}_{1}$, ..., ${Z}_{20}$) as features.

For visualization purposes, we first trained a latent space model with a two-dimensional latent space. Embedding the 31K sequences from its MSA (Fig. 4a) shows that the latent space representation of these sequences has a similar star structure as observed in Fig. 2e (Fig. 4a is the same figure as Fig. 2b. It is repeated here to be compared with Fig. 4b.). Comparing the latent space representation of sequences from the MSA (Fig. 4a) with that of chimeric sequences (Fig. 4b), we can see that the 6561 chimeric sequences, made by all possible recombinations of 3 proteins at 7 crossover locations, only occupy a small fraction of latent space available for the protein family. This suggests that most of the sequence space of cytochrome P450 is not covered by these chimeric sequences. Therefore, the two-dimensional latent space representation, though simple, is useful to estimate how much sequence space has been covered by a set of sequences. In addition, it can also potentially guide designing sequences from the unexplored sequence space by converting points in the unexplored latent space region into sequences using the VAE decoder.

Embedding the sequences that have ${T}_{50}$ data into the two-dimensional latent space and coloring the sequences based on their fitness values provide a way to visualize the fitness landscape (Fig. 4c). As the fitness landscape is not necessarily linear, Gaussian processes (GPs) are used to fit a continuous fitness surface using the two-dimensional latent space representation as features and using the radial basis function (RBF) kernel with Euclidean distance. The 278 sequences with ${T}_{50}$ experimental data are randomly separated into a training set of 222 sequences and a testing set of 56 sequences (Supplementary Table 1). Based on 10-fold cross-validation on the training set, just using the two-dimensional latent space representation of sequences, which have 466 amino acids, the GP model predicts the ${T}_{50}$ values for the training set with a Pearson correlation coefficient of $0.80\pm 0.06$ and a MAD (mean absolute deviation) of $3.1\pm 0.4$ °C (Fig. 4d). For the testing set, the Pearson correlation coefficient is 0.84 and the MAD is 2.9 °C (Fig. 4e).

As the method is not restricted to two-dimensional latent spaces, models with latent spaces of different dimensionality combined with GPs may also be used to predict the ${T}_{50}$ experimental data. Models with a latent space of dimensionality of 10, 20, 30, 40, and 50 were tested. Their performance on test set is shown in Supplementary Fig. 13. Based on 10-fold cross-validation, the model with a 20-dimensional latent space works the best, yielding a Pearson correlation coefficient of $0.97\pm 0.02$ and a MAD of $1.2\pm 0.2$ °C on the training set (Supplementary Fig. 14). On the testing set, the Pearson correlation coefficient is 0.95 and the MAD is 1.7 °C (Fig. 4f).

We note that GPs have been used before to learn the ${T}_{50}$ fitness landscape of cytochrome P450 either employing sequences as features with a structure based kernel function^56^ or using embedding representations^58^. In the study^56^ using a structure based kernel function, the Pearson correlation coefficient is 0.95 and 0.82 for two sets of testing sequences, respectively, and the MAD is 1.4 and 2.6 °C, respectively. Although our proposed method is comparable to previous methods^56,58^ in terms of prediction accuracy, our method has important differences and advantages compared to previous methods. One difference is the embedding method. The embedding method used in this study is the VAE encoder learned by modeling the sequence distribution of the protein family. Therefore, it utilizes information specific to the protein family. In contrast, the embedding method proposed in ref. ^58^ is a generic *doc2vec* embedding method, which is learned by pooling sequences from many protein families together and viewing all protein sequences equally. Another advantage of our method is that points in the embedding space, i.e., the latent space, can be converted into sequences using the VAE decoder. Therefore, the transformation between sequence space and embedding space is a two-way transformation, instead of one way as in ref. ^58^. This enables our approach to be used to propose new sequences for experimental testing based on the fitness landscape in the latent space.

### Protein stability shapes evolution

With a protein family’s MSA as training data, latent space models trained using VAEs learn the joint distribution of latent space variables ${\bf{Z}}$ and sequence variables ${\bf{X}}$: ${p}_{{\boldsymbol{\theta }}}({\bf{X}},{\bf{Z}})$. After learning a latent space model, a marginal probability ${p}_{{\boldsymbol{\theta }}}({\bf{X}})$ can be calculated for each sequence ${\bf{X}}$ with $L$ positions as ${p}_{{\boldsymbol{\theta }}}({\bf{X}})=\int {p}_{{\boldsymbol{\theta }}}({\bf{X}},{\bf{Z}}){\rm{d}}{\bf{Z}}$. The marginal probability of a sequence ${\bf{X}}$, ${p}_{{\boldsymbol{\theta }}}({\bf{X}})$, measures how likely it is that the given sequence ${\bf{X}}$ belongs to the protein family, i.e., how similar the given sequence is to the sequences from the protein family’s MSA. Because the protein family’s MSA are results of selection in protein evolution, sequences with higher probability of belonging to the protein family’s MSA are expected to have better adaptation under selection pressures. Selection pressures for protein evolution may include stability, enzyme activity, drug resistance, or other properties. It can also be a mixture of different selection pressures. Although different protein families might be under different sets of selection pressures in evolution, a common selection pressure shared by many structured protein families is protein stability. Therefore, protein stability is one of the multiple forces in shaping protein evolution and is expected to have an effect in shaping protein family sequence distribution.

One way to quantify the importance of stability in shaping protein evolution processes is calculating the correlation between stability and probabilities of protein sequences. If the evolution of a protein family is largely driven by stability, more stable sequences are more likely to be selected, i.e., have higher probability. To calculate the correlation between a protein sequence’s probability assigned by latent space models and the sequence’s stability, we utilized models learned from the two protein families: fibronectin type III domain and staphylococcal nuclease. These two protein families were used because there are both experimental data on stability change upon mutations^59^ and a large number of sequences in their MSAs in the Pfam database^2^. Because the experimental data are protein stability change between sequences that are different by one amino acid instead of the stability of an individual sequence, correlation is calculated between protein sequence stability change upon mutations and the change of probabilities assigned by the latent space model. To be comparable with experimental folding free energies, probabilities of sequences, ${p}_{{\boldsymbol{\theta }}}({\bf{X}})$, are transformed into unitless free energies by $\Delta {G}_{{\rm{VAE}}}$(${\bf{X}}$) = $-{\mathrm{log}}\ {p}_{{\boldsymbol{\theta }}}$(${\bf{X}}$), which will be called VAE free energies henceforth. The change of probabilities between sequence ${\bf{X}}$ and ${\bf{X}}^{\prime}$ is quantified by the change of VAE free energies, which is calculated as $\Delta \Delta {G}_{{\rm{VAE}}}=\Delta {G}_{{\rm{VAE}}}({\bf{X}}^{\prime})-\Delta {G}_{{\rm{VAE}}}({\bf{X}})$.

**Fig. 5:**
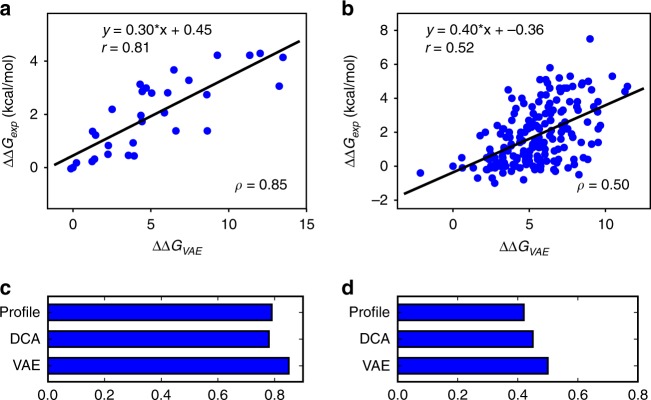
Predicting protein stability change upon mutations. **a**, **b** Correlation between experimental stability change and VAE free energy change upon single-site mutations for fibronectin type III domain (**a**) and staphylococcal nuclease (**b**). $\Delta \Delta {G}_{\exp }$ is experimental protein folding free energy change upon single-site mutations compared with the wild type protein. $\Delta \Delta {G}_{{\rm{VAE}}}$ is VAE free energy change upon single-site mutations. $\Delta \Delta {G}_{{\rm{VAE}}}$ is calculated as the change of negative log-likelihood of sequences when single-site mutations are introduced. Therefore, $\Delta \Delta {G}_{{\rm{VAE}}}$ is an unitless quantity. Each point corresponds to a mutant sequence with one mutation compared with the wild-type sequence. $r$ and $\rho$ are Pearson’s correlation coefficients and Spearman’s rank correlation coefficients, respectively. **c**, **d** In addition to latent models trained with VAEs, similar analysis is conducted using sequence profile and DCA methods. Spearman’s rank correlation coefficients between experimental protein folding free energy change upon single-site mutations and free energy change calculated using the three methods are compared for the same two protein families: fibronectin type III domain (**c**) and staphylococcal nuclease (**d**).

The Pearson’s correlation coefficients between the experimental stability change and the VAE free energy change for fibronectin type III domain and staphylococcal nuclease are 0.81 and 0.52, respectively (Fig. 5a, b and Supplementary Table 2). The corresponding Spearman’s rank correlation coefficients are 0.85 and 0.50, respectively. We note that, although the stability change of sequences correlates with their VAE free energy change, the correlation is not perfect, which supports the idea that thermal stability is only one part of the forces that drive protein evolution. For the two protein families studied here, the correlations are different, which shows that the importance of thermal stability in shaping protein evolution varies among different protein families. In addition to latent space models, similar analysis as in Fig. 5a, b is conducted using sequence profile and DCA methods. The results from the latent space models are comparable to those from both methods in terms of Spearman’s rank correlation coefficients (Fig. 5c, d).

Although protein evolution processes are only partially shaped by protein thermal stability, the correlation between protein stability change upon single-site mutations and free energy change calculated using latent space models still makes the latent space model a useful tool to predict protein stability change upon single-site mutations. The similar performance of all the three methods (Fig. 5c, d) implies that the effect of single-site mutations on protein stability can be captured as well by the simple sequence profile method as the more complicated DCA and latent space models, although the sequence profile ignores the dependency between protein positions. Because both DCA and latent space models are designed to capture dependency between protein positions, the advantage of DCA and latent space models over sequence profile might become more obvious when modeling the effect of multiple-site mutations on protein stability change, which will be further investigated in future studies.

## Discussion

Using both simulated and experimental data, we have demonstrated that latent space models, trained using VAEs and with information contained within MSAs of protein families, can capture phylogenetic relationships including ancestral relationships between sequences, help navigate protein fitness landscapes, and predict protein stability change upon single-site mutations. We note that our conclusions are robust to reasonable changes in the architecture of the artificial neural networks used in both encoder and decoder models. Setting the number of hidden units from 100 to 150 and 200 or changing the number of hidden layers from 1 to 2 does not substantially change the results (Supplementary Fig. 15). The star structure of sequences in latent space is still observed and the recapitulation of phylogenetic relationships between sequences persists.

The comparison between the phylogenetic tree structure and the latent space representation of sequences demonstrates that the latent space representation encodes similar phylogenetic relationships between sequences as does the phylogenetic tree. Phylogenetically close sequences are clustered spatially together as spikes in the latent space. In addition, as a sequence evolves, its latent space representation moves from the origin toward the outside along a spike. Quantitative comparison with the phylogeny reconstruction software FastTree 2 shows that the latent space representation is better at capturing low-resolution phylogenetic relationships and does not capture high-resolution phylogenetic relationships as well as FastTree 2. This could be because of the difference between the approximate-maximum-likelihood method implemented in FastTree 2 and our latent space models. The state-of-the-art phylogenetic inference methods, such as maximum likelihood methods, typically involve explicit mechanistic modeling of the sequence evolution process in nature. Specifically, each amino acid site is independently modeled and contributes to the likelihood function. Such modeling can be consistently powerful when divergence among sequences is relatively short. However, given long evolution time, multiple substitutions can happen at the same site, and meanwhile identical but independent substitutions can happen on different branches in the tree. Such sequence convergence can muffle the phylogenetic signals mentioned above in a large and deep phylogeny, confusing the resolution of deep branches by likelihood methods. On the contrary, latent space models consider the entire protein sequence as a whole, potentially more resistant to such loss of single-site phylogenetic patterns. Hence, latent space models can be better at capturing global structures in the sequence distribution, while some details of phylogenetic relationship might be lost in the embedding. Apart from the difference in capturing phylogenetic relationships, compared with traditional phylogenetic trees, latent space models do not require choosing a specific evolutionary model. Moreover, latent space models can work with a much larger number of sequences (hundreds of thousands of sequences or more with the computational cost increasing linearly with the number of sequences) than phylogeny reconstruction, because it does not require the tree structure search. Therefore, latent space models and phylogeny reconstruction methods are complementary and a mixture model of both phylogenetic trees and latent space models trained with VAEs might provide the best of both approaches for studying protein evolution.

When experimental data on protein fitness is available for a subset of sequences, latent space models can also help learn fitness landscapes with the low-dimensional continuous latent space representation of sequences. With an estimated fitness landscape in the latent space and the two-way transformation between the latent space and the sequence space, the latent space models can not only predict fitness values of new sequences but also help design new candidate sequences with desired fitness for experimental synthesis and testing.

With the advance of sequencing technology, the amount of protein sequence data that are available to train latent space models is increasing rapidly. Moreover, recent deep mutational scanning experiments are generating large-scale data sets of the relationship between protein sequences and function^60^. With this increasing amount of both protein sequence and fitness data, the latent space model will be a useful tool to learn information about protein evolution, fitness landscapes, and stability and provide insights into the engineering of proteins with modified properties.

Finally, we note that several other groups have also explored the use of latent space models on protein sequences^37–39^. In both refs. ^37 and 39^, the major focus is predicting mutation effects using the latent space model probability. It is similar to the part of our work on predicting protein stability change upon mutations and both yield a similar result, that the prediction from the latent space model is slightly better than the sequence profile (independent) model and DCA. It was argued in ref. ^37^ that the slightly better performance of the latent space model over DCA is because the latent space model can capture higher-order epistasis. However, compared with DCA, more domain-specific knowledge and engineering efforts were applied to the latent space model, such as the structured parameterization of the network motivated by biological priors and learning an ensemble of latent space models with a Bayesian approach. This domain-specific knowledge and ensemble-based prediction could also contribute to the better performance of the latent space model. As mentioned in ref. ^37^, the largest improvement of the latent space model’s performance seemed to result from the use of variational Bayes to learn distributions over weights of the network. Without the domain-specific knowledge and ensemble-based prediction, results in ref. ^39^ seemed to imply that the latent space model is not better than DCA in predicting effects of mutations when the number of sequences is small and is slightly better when the number of sequences is large. Similar to ref. ^39^, domain-specific knowledge and ensemble-based prediction was not used in this study. The simpler latent space model with fewer built-in assumptions used in this study could provide a more objective test of the nature of the latent space model learned using VAEs. Our findings suggest that the latent space model mostly captures the phylogenetic relationships/correlations via the latent space representation, which was not investigated in previous studies. Although the work in ref. ^38^ also used latent space models trained with VAEs, its main focus was to reduce the initial sequence search space when designing new protein sequences that have specific metal-binding sites or a structure topology. The other unique focus of our work is learning the protein fitness landscape in the latent space, which is not present in previous studies^37–39^.

## Methods

### Processing and weighting sequences in MSAs

Before being used as training data for learning latent space models, natural protein sequences in MSAs are processed to remove positions at which too many sequences have gaps and sequences with too many gaps. The processing procedure is as the following: (i) positions at which the query sequence has gaps are removed; (ii) sequences with the number of gaps >20% of the total length of the query sequence are removed; (iii) positions at which >20% of sequences have gaps are removed again; (iv) duplicated sequences are removed.

To reduce redundancy and emphasize diversity, sequences in a protein MSA are weighted. Weighting sequences can also reduce the bias in the distribution of species present in the MSA because some species’ genomes are more likely to have been sequenced than others. Although there are more complex weighting methods that reduce the influence of phylogeny^27,61,62^, here we use the simple but effective position-based sequence weights^63^ as follows. Let us represent an MSA with $N$ sequences and $L$ positions as $\{{s}_{j}^{n}:n=1...N,j=1...L\}$, where ${s}_{j}^{n}$ represents the amino acid type of the $n$th sequence at the $j$th position. In the position-based sequence weighting method^63^, the weight of a sequence is a sum of weights of the sequences’ positions. To calculate the weights of sequences, we first calculate a weight matrix $\{{w}_{j}^{n}:n=1...N,j=1...L\}$, where ${w}_{j}^{n}$ is the weight of the $n$th sequence contributed by its $j$th position. ${w}_{j}^{n}$ is calculated as

3 $${w}_{j}^{n}=\frac{1}{{C}_{j}}\times \frac{1}{{C}_{j}^{n}},$$

where ${C}_{j}$ is the number of unique amino acid types at the $j$th position of the MSA and ${C}_{j}^{n}$ is the number of sequences in the MSA that has the same amino acid type at the $j$th position as the $n$th sequence. Then the weight of the $n$th sequence is the sum of its position weights, i.e., ${w}^{n}={\sum }_{j=1}^{L}{w}_{j}^{n}$. Finally, the weights are renormalized as ${\widetilde{w}}^{n}={w}^{n}/{\sum }_{i=1}^{N}{w}^{i}$ such that the sum of the normalized weights ${\widetilde{w}}^{n}$ is one.

The above sequence processing and weighting procedure is only applied to MSAs of natural protein families. For a simulated MSA, all its sequences and positions are used and sequences are assigned with the same weight. Weights of sequences are taken into account in learning all the models presented in this study including latent space models, sequence profiles, and DCA.

### Inferring phylogenetic trees and ancestral sequences

Because the three natural protein families (fibronectin type III, cytochrome P450, and staphylococcal nuclease) have a large number of sequences in their MSAs, their phylogenetic trees were inferred using the software FastTree2^24^ with the option -lg for using the LG substitution model^47^ and the option -gamma for rescaling evolutionary lengths to optimize the Gamma20 likelihood. All three inferred phylogenetic trees are rooted using out-group rooting. Based on the phylogenetic trees inferred by FastTree2, ancestral sequences were inferred using RAxML v8.2^22^ with the option -m PROTGAMMALG to also use the LG substitution model and Gamma model of rate heterogeneity.

### Learning latent space models with VAEs

The prior distribution of ${\bf{Z}}$, ${p}_{{\boldsymbol{\theta }}}({\bf{Z}})$, is an $m$-dimensional Gaussian distribution with mean at the origin and variance initiated as the identity matrix. The decoder model ${p}_{{\boldsymbol{\theta }}}({\bf{X}}| {\bf{Z}})$ is parameterized using a fully connected artificial neural network with one hidden layer as ${\bf{H}}\ =\ \tanh ({{\bf{W}}}^{(1)}{\bf{Z}}+{{\bf{b}}}^{(1)})$ and ${p}_{{\boldsymbol{\theta }}}({\bf{X}}| {\bf{Z}})={\rm{softmax}}({{\bf{W}}}^{(2)}{\bf{H}}+{{\bf{b}}}^{(2)})$, where the parameters ${\boldsymbol{\theta }}$ include the weights $\{{{\bf{W}}}^{(1)},{{\bf{W}}}^{(2)}\}$ and the biases $\{{{\bf{b}}}^{(1)},{{\bf{b}}}^{(2)}\}$. The encoder model ${q}_{{\boldsymbol{\phi }}}({\bf{Z}}| {\bf{X}})$ is chosen to be an $m$-dimensional Gaussian distribution ${\mathcal{N}}({\boldsymbol{\mu }},{\boldsymbol{\Sigma }})$, where ${\mathbf{\Sigma }}$ is a diagonal matrix with diagonal elements of ${{\boldsymbol{\sigma }}}^{{\bf{2}}}=({\sigma }_{1}^{2},{\sigma }_{2}^{2},...,{\sigma }_{m}^{2})$. The mean ${\boldsymbol{\mu }}$ and the variance ${{\boldsymbol{\sigma }}}^{{\bf{2}}}$ are parameterized using an artificial neural network with one hidden layer as ${\bf{H}}=\tanh ({{\bf{W}}}^{(3)}{\bf{X}}+{{\bf{b}}}^{(3)})$, ${\boldsymbol{\mu }}={{\bf{W}}}^{(4)}{\bf{H}}+{{\bf{b}}}^{(4)}$, and $\mathrm{log}\,{{\boldsymbol{\sigma }}}^{{\bf{2}}}={{\bf{W}}}^{(5)}{\bf{H}}+{{\bf{b}}}^{(5)}$. The parameters ${\boldsymbol{\phi }}$ for the encoder model ${q}_{{\boldsymbol{\phi }}}({\bf{Z}}| {\bf{X}})$ include the weights $\{{{\bf{W}}}^{(3)},{{\bf{W}}}^{(4)},{{\bf{W}}}^{(5)}\}$ and the biases $\{{{\bf{b}}}^{(3)},{{\bf{b}}}^{(4)},{{\bf{b}}}^{(5)}\}$. The hidden layer is chosen to have 100 hidden units in both the encoder and the decoder models.

The weights of sequences in a protein MSA are calculated using the position-based sequence weighting^63^ shown above. Given weighted protein sequences, the parameters of both encoder and decoder models are simultaneously learned by optimizing the ELBO function^34^. To reduce overfitting, a regularization term of $\gamma \cdot {\sum }_{i=1}^{5}{\parallel {{\bf{W}}}^{(i)}\parallel }_{{\rm{F}}}^{2}$ is added to the objective ${\rm{ELBO}}({\boldsymbol{\theta }},{\boldsymbol{\phi }})$, where $\gamma$ is called the weight decay factor and $\parallel {{\bf{W}}}^{(i)}{\parallel }_{{\rm{F}}}$ is the Frobenius norm of weight matrix ${{\bf{W}}}^{(i)}$. The gradient of ELBO plus the regularization term with respect to the model parameters is calculated using the backpropagation algorithm^64^ and the parameters are optimized using the Adam optimizer^65^. The weight decay factor $\gamma$ is selected from the set of values {0.0001, 0.0005, 0.001, 0.005, 0.01, 0.05, 0.1} using 5-fold cross-validation (using 10-fold cross-validation in the case of cytochrome P450s). In the cross-validation, models trained with different weight decay factors are evaluated based on the marginal probability assigned by the model on the held-out sequences (based on the Pearson correlation coefficient in the case of cytochrome P450s).

### Calculating the marginal probability

Given a sequence ${\bf{X}}$, the marginal probability, ${p}_{{\boldsymbol{\theta }}}({\bf{X}})$, is equal to the integral $\int {p}_{{\boldsymbol{\theta }}}({\bf{X}},{\bf{Z}}){\rm{d}}{\bf{Z}}$, which is calculated using importance sampling:

4 $${p}_{{\boldsymbol{\theta }}}({\bf{X}})=	 \int {p}_{{\boldsymbol{\theta }}}({\bf{X}},{\bf{Z}}){\rm{d}}{\bf{Z}}=\int {q}_{{\boldsymbol{\phi }}}({\bf{Z}}| {\bf{X}})\frac{{p}_{{\boldsymbol{\theta }}}({\bf{X}},{\bf{Z}})}{{q}_{{\boldsymbol{\phi }}}({\bf{Z}}| {\bf{X}})}{\rm{d}}{\bf{Z}}\\ =	 \, \mathop{{\mathbb{E}}}_{{\bf{Z}} \sim {q}_{{\boldsymbol{\phi }}}({\bf{Z}}| {\bf{X}})}\left[\frac{{p}_{{\boldsymbol{\theta }}}({\bf{X}},{\bf{Z}})}{{q}_{{\boldsymbol{\phi }}}({\bf{Z}}| {\bf{X}})}\right]=\frac{1}{N} \sum \limits_{i=1}^{N}\left[\frac{{p}_{{\boldsymbol{\theta }}}({\bf{X}},{{\bf{Z}}}^{i})}{{q}_{{\boldsymbol{\phi }}}({{\bf{Z}}}^{i}| {\bf{X}})}\right],$$

where ${{\bf{Z}}}^{i}$ are independent samples from the distribution ${q}_{{\boldsymbol{\phi }}}({\bf{Z}}| {\bf{X}})$ and $N$ is number of samples. In this study, $N=1\times 1{0}^{6}$.

### Simulating MSAs

A random phylogenetic tree with 10,000 leaf nodes was generated using the *populate* function of the master Tree class from the ETE Toolkit^48^. The random branch range is chosen to be from 0 to 0.3. The LG evolutionary model^47^ was used to simulate the sequence evolution on the generated phylogenetic tree. Sequences from leaf nodes were combined into an MSA. All simulated sequences have 100 amino acids.

When exploring the position of the root node sequences in the latent space, we also considered sequences simulated with heterotachy. The sequences with heterotachy are simulated as follows. A random phylogenetic tree, T, with 10,000 leaf nodes was generated similarly as the above. Then two trees, T1 and T2, were generated based on the tree T. Both T1 and T2 have the same tree topology as T. The length of each branch in T1/T2 is set to the corresponding branch length in T multiplied by a random number that is uniformly distributed between 0 and 2. Two MSAs, each of which has 50 amino acids, were simulated based on T1 and T2, respectively. Finally, the two MSAs are concatenated into 1 MSA with 100 amino acids.

### Sequence profiles

Given a protein family’s MSA, sequence profiles^66^ model its sequence distribution by assuming protein positions are independent, i.e.,

5 $$P({\bf{S}}=({s}_{1},{s}_{2},...,{s}_{L}))=\prod _{j=1}^{L}{P}_{j}({s}_{j}),$$

where ${s}_{i}\in \{0,1,2,...,20\}$; ${s}_{j}$ represents the amino acid type (labeled using numbers from 0 to 20) at the $j$th position of the protein; and ${P}_{j}(k)$ represents the probability that the amino acid type at the $j$th position is $k$. Therefore, a profile model of a protein family with $L$ amino acids contains $21\times L$ parameters, which are ${P}_{j}(k),j=1,...,L,k=0,...,20$. These parameters are estimated using the protein family’s MSA:

6 $${P}_{j}(k)=\frac{{\sum }_{n=1}^{N}{\widetilde{w}}^{n}* I({s}_{j}^{n}=k)}{{\sum }_{n=1}^{N}{\widetilde{w}}^{n}},$$

where $N$ is the total number of sequences in the MSA; ${\widetilde{w}}^{n}$ is the normalized weight of the $n$th sequence; ${s}_{j}^{n}$ is the amino acid type at the $j$th position in the $n$th sequence of the MSA; $I({s}_{j}^{n}=k)$ is equal to 1 if ${s}_{j}^{n}=k$ and 0 otherwise. With the estimated parameters, the profile assigns a probability for any given sequence $S$ with $L$ amino acids based on Eq. (5). The free energy of the sequence is calculated as $\Delta {G}_{{\rm{Profile}}}({\bf{S}})=-{\mathrm{log}}\,P({\bf{S}})$.

### Direct coupling analysis

The DCA method^7,26–31^ models the probability of each sequence as

7 $$P({\bf{S}}=({s}_{1},{s}_{2},...,{s}_{L}))=\frac{1}{Z}\exp \left(-\left[\sum _{i=1}^{L-1}\sum _{j=i+1}^{L}{{\bf{J}}}_{ij}({s}_{i},{s}_{j})+\sum _{i=1}^{L}{{\bf{b}}}_{i}({s}_{i})\right]\right),$$

where the partition function $Z$ is

8 $$Z=\sum _{{s}_{1},{s}_{2},...,{s}_{L}}\exp \left(-\left[\sum _{i=1}^{L-1}\sum _{j=i+1}^{L}{{\bf{J}}}_{ij}({s}_{i},{s}_{j})+\sum _{i=1}^{L}{{\bf{b}}}_{i}({s}_{i})\right]\right).$$

The parameters in DCA include the bias term ${{\bf{b}}}_{i}(\cdot)$ for the $i$th position and the interaction term ${{\bf{J}}}_{ij}(\cdot,\cdot)$ between the $i$th and the $j$th position of the protein. Learning these parameters by maximizing likelihood of the model on training data involves calculating the partition function $Z$, which is computationally expensive. Therefore, the pseudo-likelihood maximization method is used to learn these parameters^26^. Similarly as in sequence profiles, the free energy of a sequence is calculated as

9 $$\Delta {G}_{{\rm{DCA}}}({\bf{S}})=-{\mathrm{log}}\,P({\bf{S}})=\sum _{i=1}^{L-1}\sum _{j=i+1}^{L}{{\bf{J}}}_{ij}({s}_{i},{s}_{j})+\sum _{i=1}^{L}{{\bf{b}}}_{i}({s}_{i})+{\mathrm{log}}\,Z.$$

Although the partition function $Z$ is not known, we can still calculate the difference of $\Delta {G}_{{\rm{DCA}}}$ between two sequences ($\Delta \Delta {G}_{{\rm{DCA}}}$), because the partition function $Z$ is a constant and does not depend on sequences.

### GP regression

GP regression method^67^ is used to fit the fitness (${T}_{50}$) landscape for chimeric cytochrome P450 sequences. To train a GP regression model, a kernel function needs to be chosen to specify the covariance between sequences^67^. When the latent space representation ${\bf{Z}}$ is used as the feature vector of sequences, the RBF kernel^67^ is used:

10 $$k({{\bf{Z}}}^{1},{{\bf{Z}}}^{2})={\sigma }_{f}^{2}\exp \left(-\frac{1}{2}\frac{| | {{\bf{Z}}}^{1}-{{\bf{Z}}}^{2}| {| }^{2}}{{\lambda }^{2}}\right),$$

where ${{\bf{Z}}}^{1},{{\bf{Z}}}^{2}$ are latent space representations of two protein sequences and $| | \cdot | |$ is the Euclidean distance in the latent space. The variance parameter ${\sigma }_{f}^{2}$ and the length scale parameter $\lambda$ in RBF are estimated by maximizing the likelihood of the GP model on ${T}_{50}$ training data. Given a test sequence ${{\bf{X}}}^{* }$, its fitness ${T}_{50}$ value is predicted as follows. First, the test sequence ${{\bf{X}}}^{* }$ is converted into the latent space representation ${{\bf{Z}}}^{* }$ using the learned encoder. Then its ${T}_{50}$ value is predicted as the expected value of the posterior distribution, i.e.,

11 $${T}_{50}({{\bf{Z}}}^{* })={{\bf{k}}}_{* }^{T}{({\bf{K}}+{\sigma }_{n}^{2}{\bf{I}})}^{-1}{\bf{y}},$$

where ${{\bf{k}}}_{* }$ is the vector of covariance between the test sequence ${{\bf{Z}}}^{* }$ and all the training sequences (${{\bf{k}}}_{* i}=k({{\bf{Z}}}^{* },{{\bf{Z}}}^{i})$); ${\bf{K}}$ is the covariance matrix between all pairs of training sequences (${{\bf{K}}}_{i,j}=k({{\bf{Z}}}^{i},{{\bf{Z}}}^{j})$). ${\sigma }_{n}^{2}$ is the variance of the experimental measure noise of ${T}_{50}$, which is also estimated by maximizing the likelihood of the GP model on the ${T}_{50}$ training data. In addition to the predicted value of ${T}_{50}$, the GP regression also provides the variance of the prediction as

12 $${\rm{var}}({T}_{50}({{\bf{Z}}}^{* }))=k({{\bf{Z}}}^{* },{{\bf{Z}}}^{* })-{{\bf{k}}}_{* }^{T}{({\bf{K}}+{\sigma }_{n}^{2}{\bf{I}})}^{-1}{{\bf{k}}}_{* }$$

### Reporting summary

Further information on research design is available in the Nature Research Reporting Summary linked to this article.

## Supplementary information

Supplementary Information

Description of Additional Supplementary Files

Supplementary Data 1

Supplementary Data 2

Supplementary Data 3

Reporting Summary

## Data Availability

The multiple sequence alignments of the three natural protein families (fibronectin type III, cytochrome P450, and staphylococcal nuclease) analyzed in this study are publicly available in the Pfam^2^ database (http://pfam.xfam.org) via Pfam accession ids (PF00041, PF00067 and PF00565). The seven realistic phylogenetic trees from the benchmark set of the FastTree study^49^ can be downloaded from the address: http://www.microbesonline.org/fasttree/downloads/aa5K_new.tar.gz. The experimental *T*_50_ values for 278 P450 sequences are downloaded from the supplementary dataset of refs. ^55,56^. The experimental folding free energies of both fibronectin type III and staphylococcal nuclease are downloaded from the Protherm database^59^.

## Code availability

The source code required to reproduce the results in this manuscript is freely available at https://github.com/xqding/PEVAE_Paper.

## References

[1] Consortium U (2018). Uniprot: the universal protein knowledgebase. Nucleic Acids Res..

[2] Finn RD (2015). The pfam protein families database: towards a more sustainable future. Nucleic Acids Res..

[3] Onuchic JN, Morcos F (2018). Protein sequence coevolution, energy landscapes and their connections to protein structure, folding and function. Biophys. J..

[4] Levy RM, Haldane A, Flynn WF (2017). Potts hamiltonian models of protein co-variation, free energy landscapes, and evolutionary fitness. Curr. Opin. Struct. Biol..

[5] Flynn WF, Haldane A, Torbett BE, Levy RM (2017). Inference of epistatic effects leading to entrenchment and drug resistance in HIV-1 protease. Mol. Biol. Evol..

[6] Figliuzzi M, Jacquier H, Schug A, Tenaillon O, Weigt M (2015). Coevolutionary landscape inference and the context-dependence of mutations in beta-lactamase TEM-1. Mol. Biol. Evol..

[7] Weigt M, White RA, Szurmant H, Hoch JA, Hwa T (2009). Identification of direct residue contacts in protein-protein interaction by message passing. Proc. Natl Acad. Sci. USA.

[8] Ortiz AR, Kolinski A, Rotkiewicz P, Ilkowski B, Skolnick J (1999). Ab initio folding of proteins using restraints derived from evolutionary information. Proteins.

[9] Skolnick J, Kolinski A, Brooks CL, Godzik A, Rey A (1993). A method for predicting protein structure from sequence. Curr. Biol..

[10] Roy A, Kucukural A, Zhang Y (2010). I-tasser: a unified platform for automated protein structure and function prediction. Nat. Protoc..

[11] Kamisetty H, Ovchinnikov S, Baker D (2013). Assessing the utility of coevolution-based residue-residue contact predictions in a sequence-and structure-rich era. Proc. Natl Acad. Sci. USA.

[12] Ovchinnikov S (2017). Protein structure determination using metagenome sequence data. Science.

[13] Ovchinnikov S (2015). Large-scale determination of previously unsolved protein structures using evolutionary information. Elife.

[14] Bueno CA, Potoyan DA, Cheng RR, Wolynes PG (2018). Prediction of changes in protein folding stability upon single residue mutations. Biophys. J..

[15] Wheeler LC, Lim SA, Marqusee S, Harms MJ (2016). The thermostability and specificity of ancient proteins. Curr. Opin. Struct. Biol..

[16] Lim SA, Hart KM, Harms MJ, Marqusee S (2016). Evolutionary trend toward kinetic stability in the folding trajectory of rnases h. Proc. Natl Acad. Sci. USA.

[17] Hart KM (2014). Thermodynamic system drift in protein evolution. PLoS Biol..

[18] Yang Z (2006). Computational Molecular Evolution.

[19] Felsenstein J (1981). Evolutionary trees from dna sequences: a maximum likelihood approach. J. Mol. Evol..

[20] Yang Z (2007). Paml 4: phylogenetic analysis by maximum likelihood. Mol. Biol. Evol..

[21] Huelsenbeck JP, Ronquist F (2001). Mrbayes: Bayesian inference of phylogenetic trees. Bioinformatics.

[22] Stamatakis A, Ludwig T, Meier H (2004). Raxml-iii: a fast program for maximum likelihood-based inference of large phylogenetic trees. Bioinformatics.

[23] Guindon S (2010). New algorithms and methods to estimate maximum-likelihood phylogenies: assessing the performance of phyml 3.0. Syst. Biol..

[24] Price MN, Dehal PS, Arkin AP (2010). Fasttree 2-approximately maximum-likelihood trees for large alignments. PLoS ONE.

[25] Sailer ZR, Harms MJ (2017). High-order epistasis shapes evolutionary trajectories. PLoS Comput. Biol..

[26] Ekeberg M, Lövkvist C, Lan Y, Weigt M, Aurell E (2013). Improved contact prediction in proteins: using pseudolikelihoods to infer potts models. Phys. Rev. E.

[27] Morcos F (2011). Direct-coupling analysis of residue coevolution captures native contacts across many protein families. Proc. Natl Acad. Sci. USA.

[28] Cocco S, Feinauer C, Figliuzzi M, Monasson R, Weigt M (2018). Inverse statistical physics of protein sequences: a key issues review. Rep. Prog. Phys..

[29] Hopf TA (2017). Mutation effects predicted from sequence co-variation. Nat. Biotechnol..

[30] Marks DS (2011). Protein 3d structure computed from evolutionary sequence variation. PLoS ONE.

[31] Ovchinnikov S, Kamisetty H, Baker D (2014). Robust and accurate prediction of residue-residue interactions across protein interfaces using evolutionary information. Elife.

[32] Balakrishnan S, Kamisetty H, Carbonell JG, Lee S-I, Langmead CJ (2011). Learning generative models for protein fold families. Proteins.

[33] Qin C, Colwell LJ (2018). Power law tails in phylogenetic systems. Proc. Natl Acad. Sci. USA.

[34] Kingma, D. P. & Welling, M. *Auto-encoding Variational Bayes* (ICLR, 2013).

[35] Rezende, D. J., Mohamed, S. & Wierstra, D. *Stochastic Backpropagation and Approximate Inference in Deep Generative Models* (ICML, 2014).

[36] Blei DM, Kucukelbir A, McAuliffe JD (2017). Variational inference: a review for statisticians. J. Am. Stat. Assoc..

[37] Riesselman AJ, Ingraham JB, Marks DS (2018). Deep generative models of genetic variation capture the effects of mutations. Nat. Methods.

[38] Greener JG, Moffat L, Jones DT (2018). Design of metalloproteins and novel protein folds using variational autoencoders. Sci. Rep..

[39] Sinai, S., Kelsic, E., Church, G. M. & Nowak, M. A. Variational auto-encoding of protein sequences. *NIPS Workshop on Machine Learning in Computational Biology* (2017).

[40] Dempster AP, Laird NM, Rubin DB (1977). Maximum likelihood from incomplete data via the em algorithm. J. R. Stat. Soc. B.

[41] Neal, R. M. & Hinton, G. E. In *Learning in Graphical Models* 355–368 (Springer, 1998).

[42] Jordan MI, Ghahramani Z, Jaakkola TS, Saul LK (1999). An introduction to variational methods for graphical models. Mach. Learn..

[43] Wainwright MJ, Jordan MI (2008). Graphical models, exponential families, and variational inference. Found. Trends Mach. Learn..

[44] Hoffman, M. D., Blei, D. M., Wang, C. & Paisley, J. Stochastic variational inference. *J. Mach. Learn. Res.* **14**, 1303–1347 (2013).

[45] Bowman, S. R. et al. Generating sentences from a continuous space. In *Proc. of The 20th SIGNLL Conference on Computational Natural Language Learning*, 10–21 (Association for Computational Linguistics, Berlin, Germany, 2016).

[46] Ravanbakhsh, S., Lanusse, F., Mandelbaum, R., Schneider, J. G. & Poczos, B. Enabling dark energy science with deep generative models of galaxy images. In *Proc. Thirty-First AAAI Conference on Artificial Intelligence* 1488–1494 (AAAI Press, 2017).

[47] Le SQ, Gascuel O (2008). An improved general amino acid replacement matrix. Mol. Biol. Evol..

[48] Huerta-Cepas J, Serra F, Bork P (2016). Ete 3: reconstruction, analysis, and visualization of phylogenomic data. Mol. Biol. Evol..

[49] Price MN, Dehal PS, Arkin AP (2009). Fasttree: computing large minimum evolution trees with profiles instead of a distance matrix. Mol. Biol. Evol..

[50] Guindon S, Gascuel O (2003). A simple, fast, and accurate algorithm to estimate large phylogenies by maximum likelihood. Syst. Biol..

[51] Ward JH (1963). Hierarchical grouping to optimize an objective function. J. Am. Stat. Assoc..

[52] Vinh NX, Epps J, Bailey J (2010). Information theoretic measures for clusterings comparison: Variants, properties, normalization and correction for chance. J. Mach. Learn. Res..

[53] Jolliffe, I. In *International Encyclopedia of Statistical Science* (ed. Lovric, M.) 1094–1096 (Springer, 2011).

[54] Maaten Lvd, Hinton G (2008). Visualizing data using t-. J. Mach. Learn. Res..

[55] Otey CR (2006). Structure-guided recombination creates an artificial family of cytochromes p450. PLoS Biol..

[56] Romero PA, Krause A, Arnold FH (2013). Navigating the protein fitness landscape with gaussian processes. Proc. Natl Acad. Sci. USA.

[57] Li Y (2007). A diverse family of thermostable cytochrome p450s created by recombination of stabilizing fragments. Nat. Biotechnol..

[58] Yang KK, Wu Z, Bedbrook CN, Arnold FH (2018). Learned protein embeddings for machine learning. Bioinformatics.

[59] Gromiha MM (2000). Protherm, version 2.0: thermodynamic database for proteins and mutants. Nucleic Acids Res..

[60] Fowler DM, Fields S (2014). Deep mutational scanning: a new style of protein science. Nat. Methods.

[61] Dunn SD, Wahl LM, Gloor GB (2007). Mutual information without the influence of phylogeny or entropy dramatically improves residue contact prediction. Bioinformatics.

[62] Burger L, Van Nimwegen E (2010). Disentangling direct from indirect co-evolution of residues in protein alignments. PLoS Comput. Biol..

[63] Henikoff S, Henikoff JG (1994). Position-based sequence weights. J. Mol. Biol..

[64] Rumelhart, D. E., Hinton, G. E. & Williams, R. J. In *Parallel Distributed Processing: Explorations in the Microstructure of Cognition* 318–362 (MIT, Cambridge, MA, 1986).

[65] Kinga, D. P. & Ba, J. Adam: a method for stochastic optimization. In *3rd International Conference for Learning Representations* (ICLR, 2015).

[66] Söding J, Biegert A, Lupas AN (2005). The hhpred interactive server for protein homology detection and structure prediction. Nucleic Acids Res..

[67] Rasmussen, C. E. Gaussian processes in machine learning. In *Advanced Lectures on Machine Learning* 63–71 (Springer, 2004).

