# Supplementary material for: Deciphering protein evolution and fitness landscapes with latent space models
Source: Nat Commun. 2019 Dec 10;10:5644. doi: 10.1038/s41467-019-13633-0 (PMC6904478; doi:10.1038/s41467-019-13633-0)
Supplement: Supplementary file 1 — Supplementary Information [file 41467_2019_13633_MOESM1_ESM.pdf]

Supplementary Information

Deciphering protein evolution and fitness landscapes with latent space models

Ding et al.

Supplementary Figures

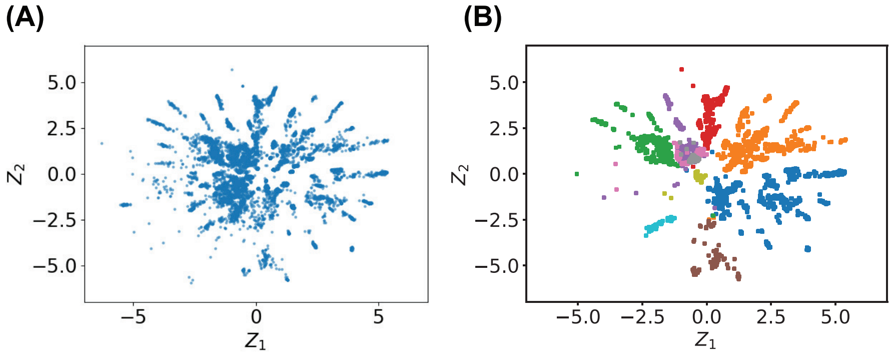

Supplementary Figure 1: **(A)** Latent space representation of sequences from the multiple sequence alignment of the staphylococcal nuclease family. **(B)** Similar plot as Fig. 2G for the staphylococcal nuclease family

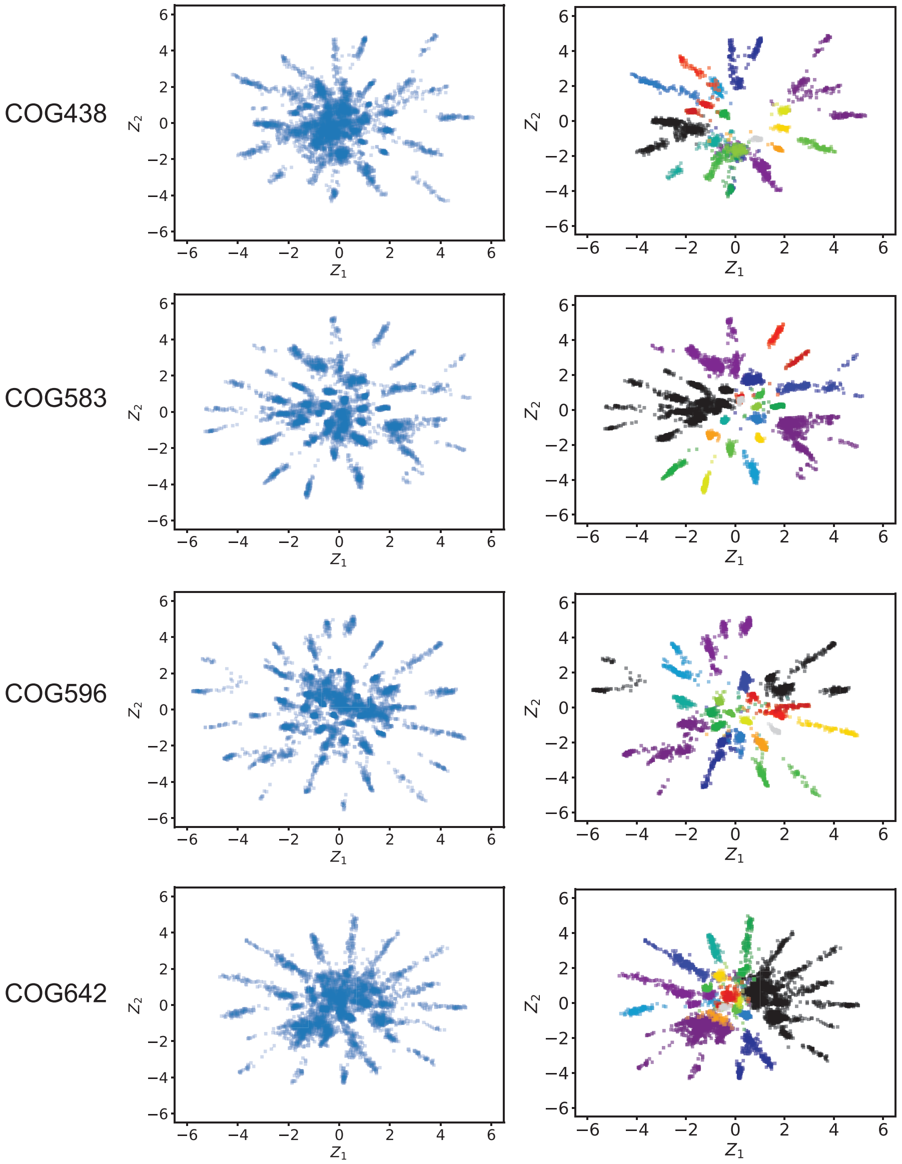

Supplementary Figure 2: Latent space representations of simulated sequences generated based on realistic phylogenetic trees of protein families. These protein families (COG438, COG583, COG596, COG642) are selected from the Clusters of Orthologous Groups (COG) database. **(Left)** Latent space representation of sequences from the simulated MSAs. **(Right)** Similar plot as Fig. 2G for COG protein families.

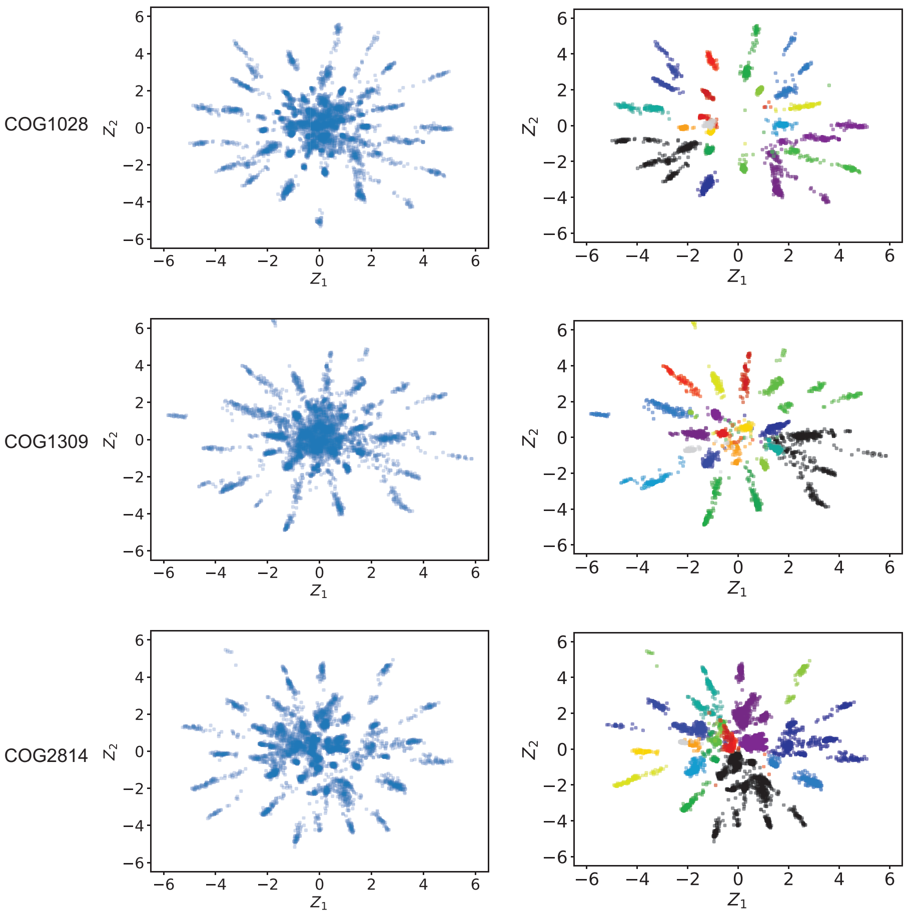

Supplementary Figure 3: Latent space representations of simulated sequences generated based on realistic phylogenetic trees of protein families. These protein families (COG1028, COG1309, COG2814) are selected from the Clusters of Orthologous Groups (COG) database. **(Left)** Latent space representation of sequences from the simulated MSAs. **(Right)** Similar plot as Fig. 2G for COG protein families.

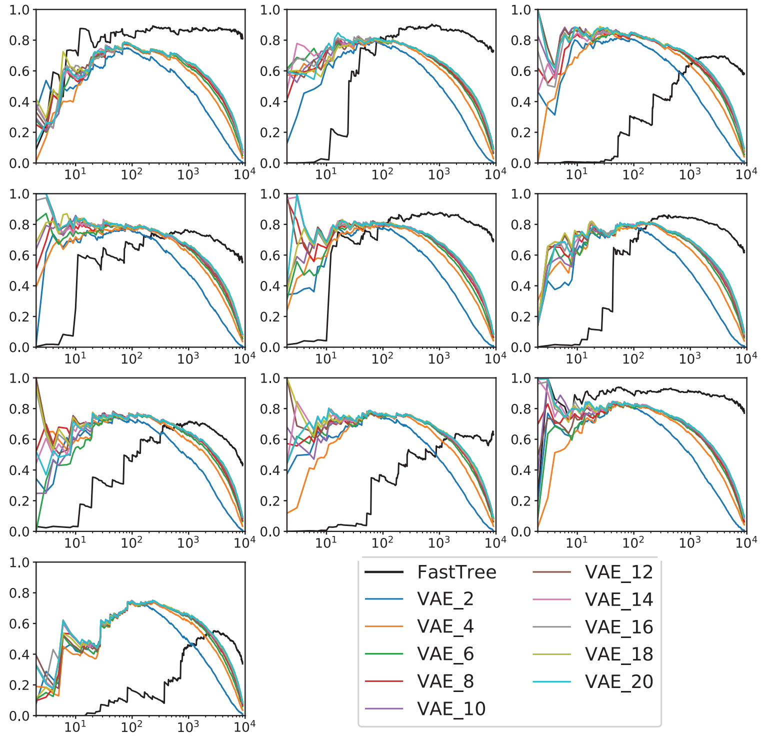

Supplementary Figure 4: The adjusted mutual information (AMI) between the true clustering and the clustering results based on FastTree 2 and the latent space representation of variational auto-encoders (VAEs) with different latent space dimensions (the dimension of latent space varies from 2 to 20). The y axis is the AMI. The x axis is the logrithm of the number of clusters. Clustering results with larger number of clusters has higher clustering resolution. The largest number of clusters, i.e., the most right point in the plots, is 8000.

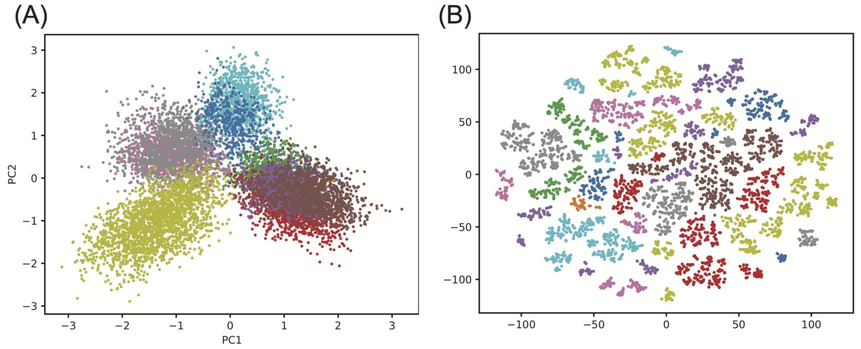

Supplementary Figure 5: The dimension reduction results of the simulated sequences from Fig.2E using **(A)** principal componenet analysis (PCA) and **(B)** t-SNE. Each point represent a simulated sequence. Points are colored similarly as in Fig. 2E.

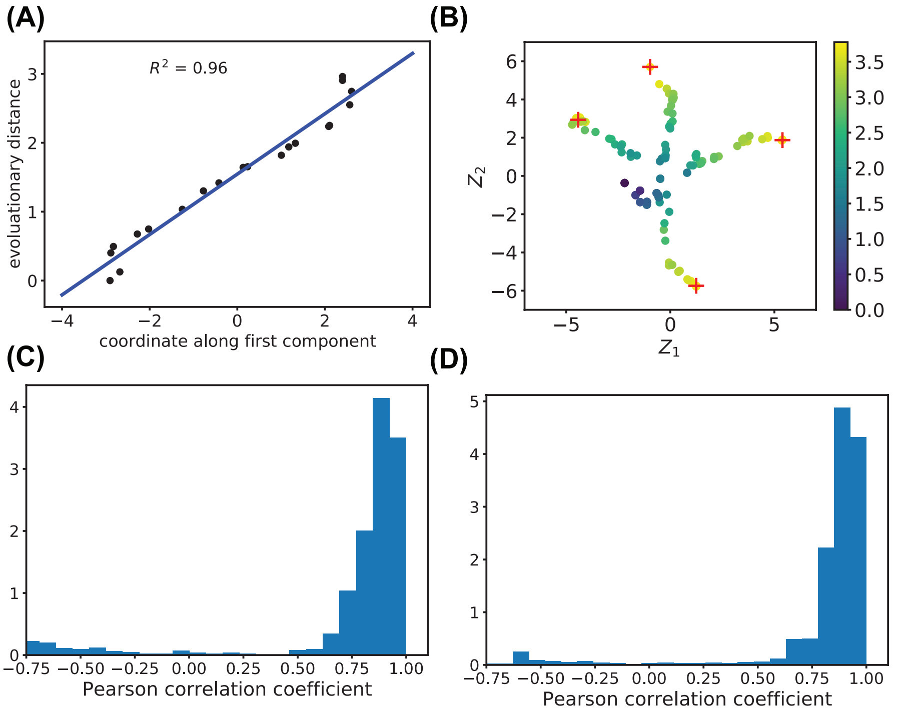

Supplementary Figure 6: **(A)** The Pearson correlation coefficient between sequences' evolutionary distances and their positions along the first component for the rightmost leaf node sequence in Fig. 3A.II and its ancestral sequences. **(B)** A similar plot as Fig. 3A.II for the staphylococcal nuclease family. **(C and D)** Similar plots as Fig. 3A.IV for the P450 family and the staphylococcal nuclease family, respectively.

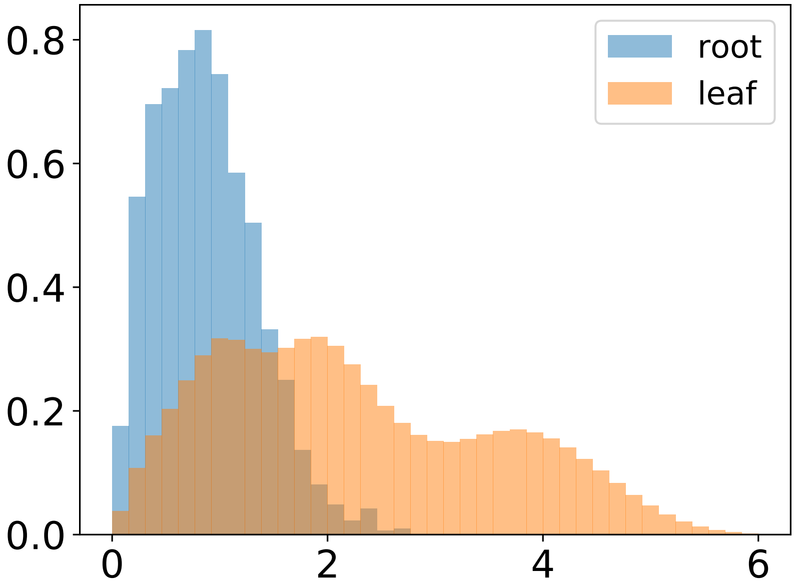

Supplementary Figure 7: Distribution of distances from the origin for the root sequences (Fig. 3A.III) and the sequences in the alignments (sequence on the leaf nodes) in the two dimensional latent space.

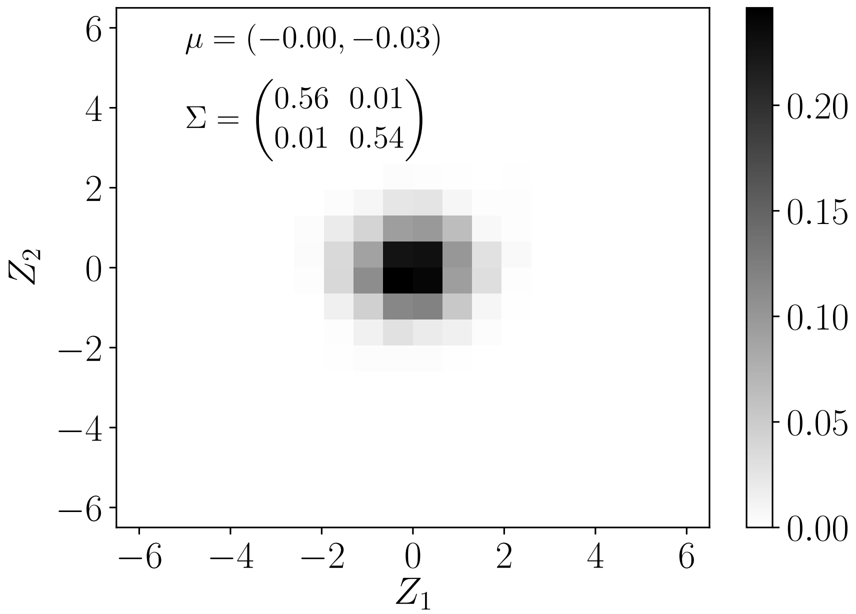

Supplementary Figure 8: A similar plot as Fig. 3A.III for simulated sequences with heterotachy.

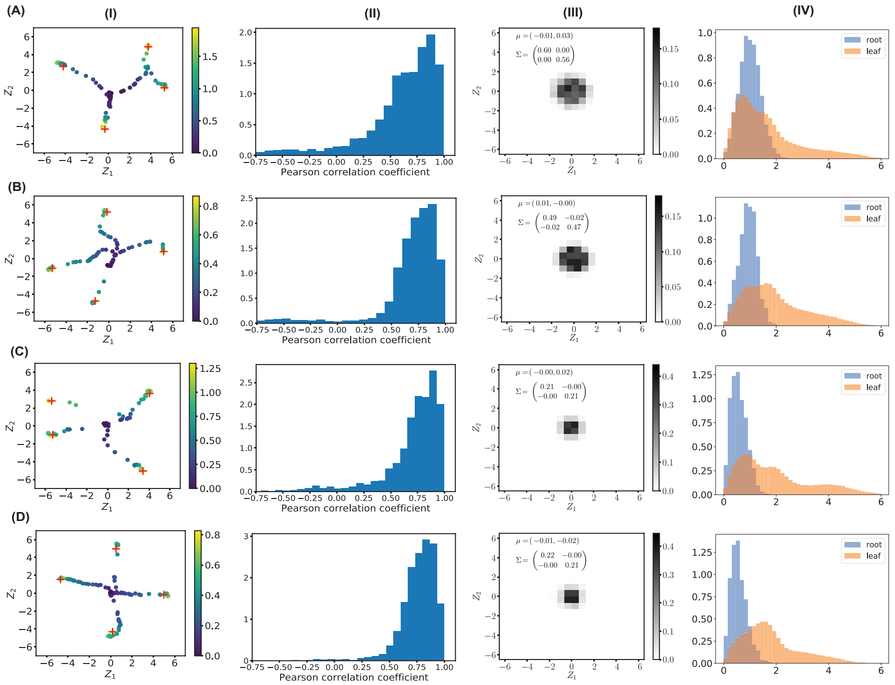

Supplementary Figure 9: Results for COG protein families: **(A)** COG438; **(B)** COG583 ; **(C)** COG596; **(D)** COG642. **(I)** Similar plots as Fig. 3A.II for COG protein families. **(II)** Similar plot as Fig. 3A.IV for COG protein families. **(III)** Similar plots as Fig. 3A.III for COG protein families. **(IV)** Distribution of distances from the origin for the root sequences and the sequences in the alignments (sequence on the leaf nodes) in the two dimensional latent space.

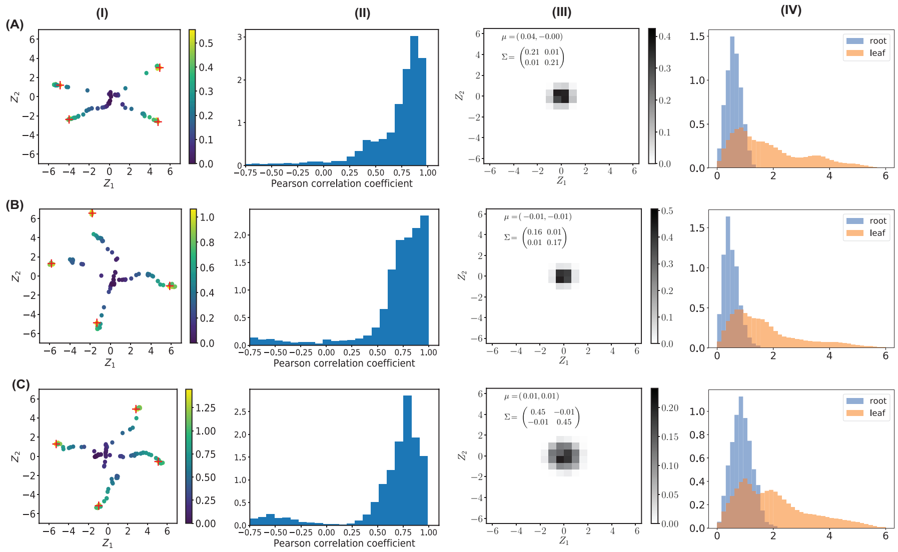

Supplementary Figure 10: Results for COG protein families: **(A)** COG1028; **(B)** COG1309 ; **(C)** COG2814. **(I)** Similar plots as Fig. 3A.II for COG protein families. **(II)** Similar plot as Fig. 3A.IV for COG protein families. **(III)** Similar plots as Fig. 3A.III for COG protein families. **(IV)** Distribution of distances from the origin for the root sequences and the sequences in the alignments (sequence on the leaf nodes) in the two dimensional latent space.

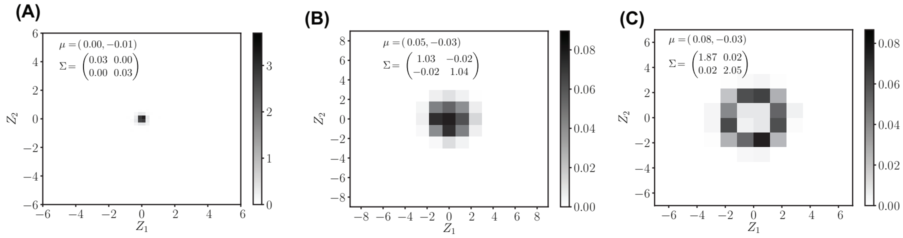

Supplementary Figure 11: **(Left)** Similar plots as Fig. 3A.III for fibronectin type III domain. **(Middle)** Similar plot as Fig. 3A.III for P450 protein family. **(Right)** Similar plots as Fig. 3A.III for staphylococcal nuclease protein family.

```
CYP102A1    .TIKEMPQPKTFGELKNLPLLNTDKPVQALMKIADELGEIFKFEAPGRVTRYLSSQRLIKEACDESRFDK
CYP102A2    KETSPIPQPKTFGPLGNLPLIDKDKPTLSLIKLAEEQGPIFQIHTPAGTTIVVSGHELVKEVCDEERFDK
CYP102A3    KQASAIPQPKTYGPLKNLPHLEKEQLSQSLWRIADELGPIFRFDFPGVSSVFVSGHNLVAEVCDEKRFDK

CYP102A1    NLSQALKFVRDFAGDGLATSWTHEKNWKKAHNILLPSFSQQAMKGYHAMMVDIAVQLVQKWERLNADEHI
CYP102A2    SIEGALEKVRAFSGDGLATSWTHEPNWRKAHNILMPTFSQRAMKDYHEKMVDIAVQLIQKWARLNPNEAV
CYP102A3    NLGKGLQKVREFGGDGLATSWTHEPNWQKAHRILLPSFSQKAMKGYHSMMLDIATQLIQKWSRLNPNEEI

CYP102A1    EVPEDMTRLTLDTIGLCGFNYRFNSFYRDQPHPFITSMVRALDEAMNKLQRANPDDPAYDENKRQFQEDI
CYP102A2    DVPGDMTRLTLDTIGLCGFNYRFNSYYRETPHPFINSMVRALDEAMHQMQRLDVQDKLMVRTKRQFRYDI
CYP102A3    DVADDMTRLTLDTIGLCGFNYRFNSFYRDSQHPFITSMLRALKEAMNQSKRLGLQDKMMVKTKLQFQKDI

CYP102A1    KVMNDLVDKIIADRKASGEQ.SDDLLTHMLNGKDPETGEPLDDENIRYQIITFLIAGHETTSGLLSFALY
CYP102A2    QTMFSLVDSIIAERRANGDQDEKDLLARMLNVEDPETGEKLDDENIRFQIITFLIAGHETTSGLLSFATY
CYP102A3    EVMNSLVDRMIAERKANPDENIKDLLSLMLYAKDPVTGETLDDENIRYQIITFLIAGHETTSGLLSFAIY

CYP102A1    FLVKNPHVLQKAAEEAARVLVDPVPSYKQVKQLKYVGMVLNEALRLWPTAPAFSLYAKEDTVLGGEYPLE
CYP102A2    FLLKHPDKLKKAYEEVDRVLTDAAPTYKQVLELTYIRMILNESLRLWPTAPAFSLYPKEDTVIGGKFPIT
CYP102A3    CLLTHPEKLKKAQEEADRVLTDDTPEYKQIQQLKYIRMVLNETLRLYPTAPAFSLYAKEDTVLGGEYPIS

CYP102A1    KGDELMVLIPQLHRDKTIWGDDVEEFRPERFENPSAIPQHAFKPFGNGQRACIGQQFALHEATLVLGMML
CYP102A2    TNDRISVLIPQLHRDRDAWGKDAEEFRPERFEHQDQVPHHAYKPFGNGQRACIGMQFALHEATLVLGMIL
CYP102A3    KGQPVTVLIPKLHRDQNAWGPDAEDFRPERFEDPSSIPHHAYKPFGNGQRACIGMQFALQEATMVLGLVL

CYP102A1    KHFDFEDHTNYELDIKETLTLKPEGFVVKAKSKKIPLGGIPSPST.
CYP102A2    KYFTLIDHENYELDIKQTLTLKPGDFHISVQSRHQEAIHADVQAAE
CYP102A3    KHFELINHTGYELKIKEALTIKPDDFKITVKPRKTAAINVQRKEQA
```

Supplementary Figure 12: Sequences of the three parent cytochrome P450s (CYP102A1, CYP102A2, CYP102A3). The chimeric sequences are made by recombining the three proteins at the seven crossover locations marked by arrows.

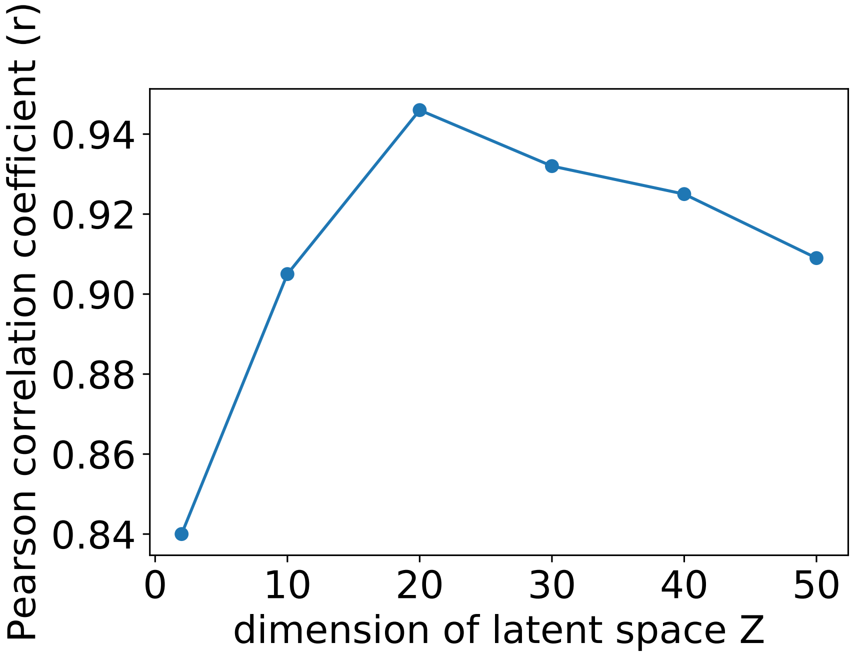

Supplementary Figure 13: Pearson correlation coefficients between the Gaussian process's prediction and experimental T_{50} data on the test set of chimeric cytochrome P450 sequences when latent spaces with different dimensions are used.

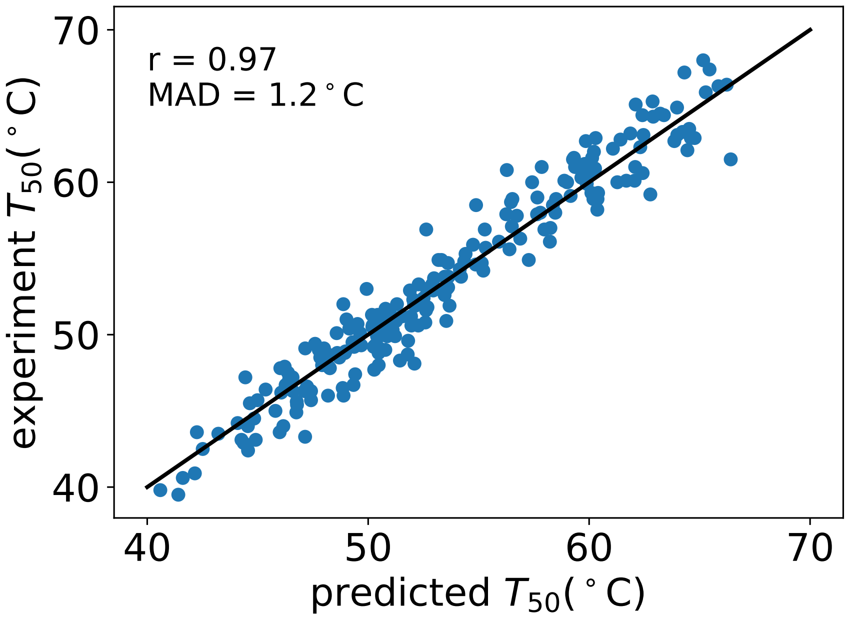

Supplementary Figure 14: The Gaussian process's performance at predicting T_{50} on the training set of 222 chimeric cytochrome P450 sequences using the 20 dimensional latent space representation $(Z_1, ..., Z_{20})$ as features and using the radial basis function kernel with Euclidean distance in the latent space $\mathbf{Z}$.

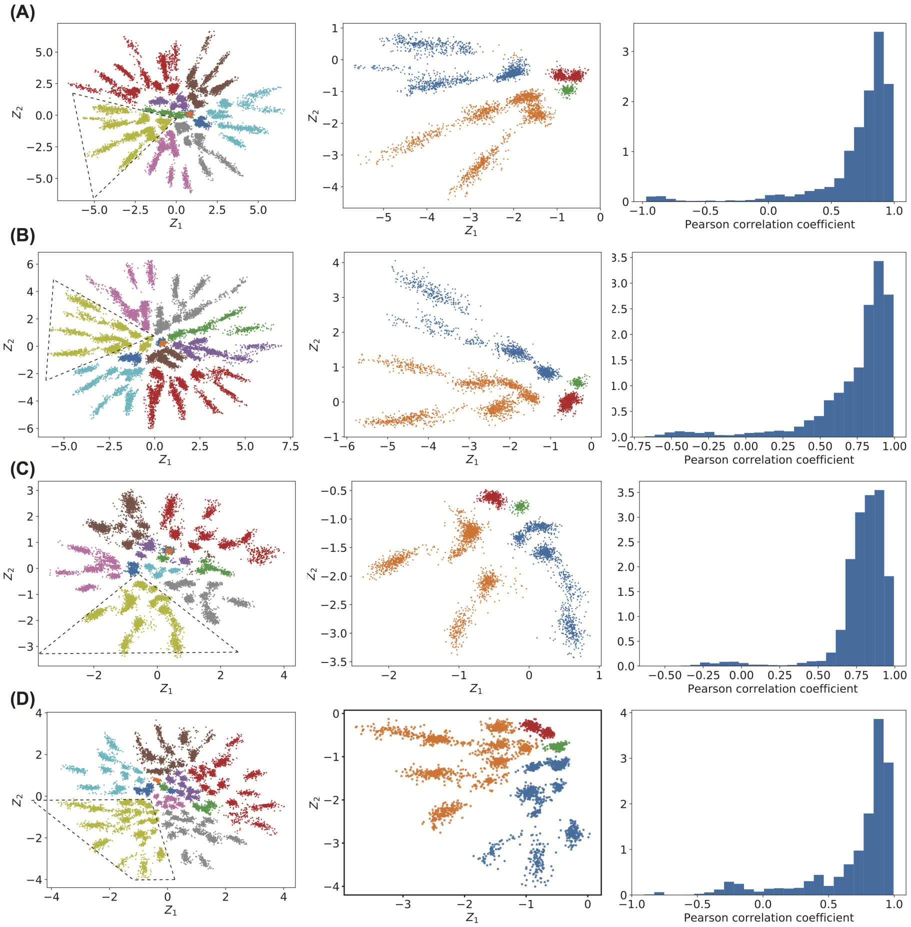

Supplementary Figure 15: Results on simulated sequences when different architectures of the artificial neuron networks are used in the encoder and decoder models. **(A)** One hidden layer with 150 nodes. **(B)** One hidden layer with 200 nodes. **(C)** Two hidden layers each of which has 50 nodes. **(D)** Two hidden layers each of which has 100 nodes. **(Left column)** Similar plots as Fig. 2.E. **(Middle column)** Similar plots as Fig. 2.F showing the latent space representations of sequences from the yellow colored group (enclosed by the dashed lines). **(Right column)** Similar plots as Fig. 3.A.IV.
