# Supplementary material for: Deciphering protein evolution and fitness landscapes with latent space models
Source: Nat Commun. 2019 Dec 10;10:5644. doi: 10.1038/s41467-019-13633-0 (PMC6904478; doi:10.1038/s41467-019-13633-0)
Supplement: Supplementary file 2 — Description of Additional Supplementary Files [file 41467_2019_13633_MOESM2_ESM.pdf]

Description of Additional Supplementary Files

File Name: Supplementary Data 1
Description: Chimeric cytochrome P450 sequences that are not made by recombining the three parent cytochrome P450s (CYP102A1, CYP102A2, CYP102A3) at the seven cross over positions in Supplementary Fig. 12.

File Name: Supplementary Data 2
Description: Experimental and predicted T50 values for chimeric cytochrome P450 sequences.

File Name: Supplementary Data 3
Description: Experimental and predicted free energy changes upon single site mutations.
